# The stroke risk gene *Foxf2* maintains brain endothelial cell function via Tie2 signaling

**DOI:** 10.1038/s41593-025-02136-5

**Published:** 2025-12-15

**Authors:** Katalin Todorov-Völgyi, Judit González-Gallego, Stephan A. Müller, Mihail Ivilinov Todorov, Fatma Burcu Seker, Simon Frerich, Filippo M. Cernilogar, Luise Schröger, Rainer Malik, Jiayu Cao, Gemma Llovera, Stefan Roth, Ulrike Schillinger, Martina Schifferer, Azadeh Reyahi, Dennis Crusius, Liliana D. Pedro, Mikael Simons, Peter Carlsson, Ali Ertürk, Arthur Liesz, Gunnar Schotta, Nikolaus Plesnila, Stefan F. Lichtenthaler, Dominik Paquet, Martin Dichgans

**Affiliations:** 1 Institute for Stroke and Dementia Research (ISD), University Hospital, LMU Munich, Munich, Germany; 2 Graduate School of Systemic Neuroscience (GSN), University Hospital, LMU Munich, Munich, Germany; 3 German Center for Neurodegenerative Diseases (DZNE), Munich, Germany; 4 Neuroproteomics, School of Medicine and Health, Klinikum Rechts der Isar, Technical University of Munich, Munich, Germany; 5 Institute for Intelligent Biotechnologies (IBIO), Helmholtz Zentrum München, Neuherberg, Germany; 6 Division of Molecular Biology, Biomedical Center, Faculty of Medicine, LMU Munich, Martinsried, Germany; 7 Department of Science and Technological Innovation, University of Piemonte Orientale, Alessandria, Italy; 8 Munich Cluster for Systems Neurology (SyNergy), Munich, Germany; 9 Department of Chemistry and Molecular Biology, University of Gothenburg, Gothenburg, Sweden; 10 Institute of Neuronal Cell Biology, Technical University of Munich, Munich, Germany; 11 German Center for Cardiovascular Diseases (DZHK), Munich, Germany

**Keywords:** Blood-brain barrier, Proteomic analysis, Stroke

## Abstract

Cerebral small vessel disease (SVD) is a common chronic cerebrovascular disorder with poorly understood pathomechanisms. Genetic studies have identified *FOXF2* as a major risk gene for both SVD and stroke. FOXF2 encodes a transcription factor primarily expressed in brain pericytes and endothelial cells (ECs); however, its mechanistic role in cerebrovascular disease remains unknown. Here we show that Foxf2 maintains EC function through Tie2 signaling. RNA and chromatin sequencing identified FOXF2 as a transcriptional activator of Tie2 and other endothelial lineage-specific genes. The deletion of EC-specific Foxf2 in adult mice resulted in blood–brain barrier leakage, which worsened after experimental stroke. Proteomic analyses of Foxf2-deficient mouse brain-derived and human-induced pluripotent stem cell-derived ECs that lack *FOXF2* revealed a downregulation of multiple proteins involved in Tie2 signaling. Endothelial Foxf2 deficiency impaired functional hyperemia, reduced NO production and increased infarct size through disrupted Tie2 signaling, effects that were rescued by pharmacological activation of Tie2 with AKB-9778. Collectively, our results highlight the critical role of Foxf2-regulated Tie2 signaling in SVD and stroke, suggesting new avenues for therapeutic interventions.

## Abstract (web summary)

We show that FOXF2 maintains cerebrovascular function through Tie2 signaling, and that pharmacological activation of Tie2 with AKB-9778 rescues deficits in cerebral small vessel disease and stroke.

## Main

Stroke is the leading cause of long-term disability and second leading cause of death^1^. Cerebral small vessel disease (SVD) accounts for up to 30% strokes and most cases of vascular dementia^2,3^, but the mechanisms underlying SVD are poorly understood. Brain endothelial cells (BECs) serve as a unique function in controlling the integrity of the blood–brain barrier (BBB), regulating cerebral blood flow (CBF) and maintaining brain homeostasis^4,5^. Studies in rodent models of SVD have pointed to a role of endothelial dysfunction^2,6–9^. Consistent with this, neuroimaging and autopsy studies in SVD patients have provided evidence of impaired cerebrovascular reactivity^2,10,11^ and loss of BBB integrity^12,13^. Endothelial dysfunction also contributes to the pathophysiology of stroke, including BBB breakdown, after cerebral ischemia^14,15^. However, the molecular pathways underlying endothelial dysfunction in these conditions are insufficiently understood^16^.

Recent genome-wide association studies (GWAS) have identified *FOXF2* as a major risk gene for stroke and SVD^17–23^. Foxf2 encodes forkhead box f2, a transcription factor that is specifically enriched in BECs compared to endothelial cells (ECs) from other organs^24–26^, suggesting a unique role of Foxf2 in brain endothelium. Interestingly, global inactivation of Foxf2 in mice results in defects of the BBB, endothelial thickening and increased *trans*-endothelial transport^27^. This phenotype has been attributed to a deficiency of Foxf2 in pericytes and a requirement for Foxf2 in pericyte differentiation^27^, but also relates to the primary function of Foxf2 in brain endothelium. In support of this, Foxf2 expression in cultured ECs has been shown to induce the expression of BBB maturation and BEC differentiation markers^24^.

EC-specific functions are secured through dedicated molecular pathways such as angiopoietin (ANG)–Tie2 signaling^28,29^ and Vegf–Vegfr2 signaling^30,31^, and through downstream effectors, including nitric oxide (NO), a key modulator of blood flow^32,33^. EC functions are further controlled by Foxo1, a key transcription factor in ECs and a major regulator of endothelial quiescence^34,35^. Foxo1 downregulates Nos3 (ref. ^36^) and Cldn5 (refs. ^37,38^) expression while upregulating Ang2 (ref. ^36^), a context-dependent antagonist of Ang1–Tie2 signaling. In turn, the activation of Tie2-PI3K/Akt signaling by Ang1 promotes Akt-mediated phosphorylation, leading to nuclear exclusion^39,40^ and subsequent degradation of Foxo1 (ref. ^36^). However, detailed studies on the role of Foxf2 in ECs are lacking, and the molecular and cellular pathways by which Foxf2 maintains EC function in vivo are still unknown. Also, the mechanisms linking Foxf2 to SVD and stroke remain unexplored.

To address these questions, we performed studies in a new mouse model with inducible deletion of Foxf2 in ECs and human-induced pluripotent stem cell (iPSC)-derived ECs (iECs) lacking *FOXF2*. We show that FOXF2 acts as a transcriptional activator of cell-adhesion-related and angiogenesis-related genes, including *Tie2*. We further find that endothelial Foxf2 maintains BEC function through Tie2 signaling and protects against manifestations of SVD and stroke. Specifically, Foxf2 stabilized the BBB both in naive animals and upon experimental stroke. We further demonstrate that endothelial Foxf2 promotes NO signaling, facilitates functional hyperemia and limits infarct size via Tie2 signaling. Pharmacological treatment with the Tie2-activator AKB-9778 rescued the effects of Foxf2 deficiency on key outcomes.

## Results

### FOXF2 acts as a transcriptional activator of cell-adhesion-related and angiogenesis-related genes including *TIE2*

**Fig. 1:**
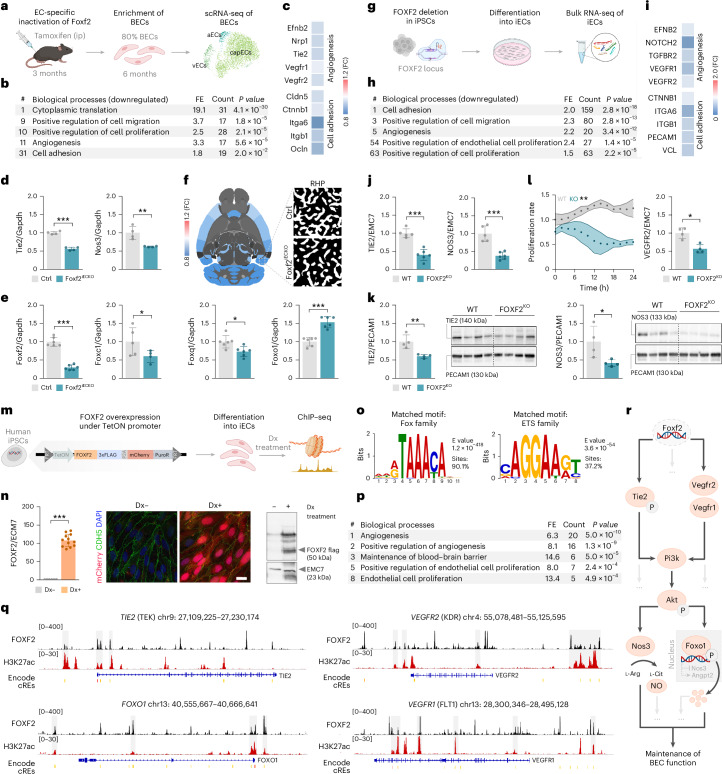
FOXF2 acts as a transcriptional activator of cell-adhesion-related and angiogenesis-related genes including *TIE2*. **a**, Experimental outline. scRNA-seq of BECs enriched from Foxf2^iECKO^ and Ctrl mouse brain. **b**, Enrichment analysis of biological processes of significantly downregulated transcripts in Foxf2^iECKO^ versus Ctrl mice. **c**, mRNA abundance of the most affected angiogenesis and cell-adhesion-related receptors in BECs of Foxf2^iECKO^ versus Ctrl mice. **d**, Relative mRNA abundance of Tie2 and Nos3 transcripts in full brain tissue of Foxf2^iECKO^ versus Ctrl mice. **e**, Relative mRNA abundance of selected FOX transcription factors in Foxf2^iECKO^ versus Ctrl mice (normalized to Gapdh and Ctrl) in the whole brain. **f**, Decreased vessel density in cortical regions of Foxf2^iECKO^ versus Ctrl mice (significantly altered brain regions are highlighted in blue). **g**, Experimental outline. Human iPSCs genome edited for FOXF2 deletion and differentiated into iECs for bulk RNA-seq. **h**, Enrichment analysis of biological processes of significantly downregulated transcripts in FOXF2^KO^ versus WT iECs. **i**, mRNA abundance of the most affected angiogenesis and cell-adhesion-related receptors in FOXF2^KO^ versus WT iECs. **j**, Relative mRNA abundance of TIE2 and NOS3 transcripts in FOXF2^KO^ versus WT iECs. **k**, Relative protein abundance of TIE2 and NOS3 in human FOXF2^KO^ versus WT iECs (normalized to PECAM1 and WT). **l**, Proliferation rate of FOXF2^KO^ versus WT iECs (left) and relative mRNA abundance of VEGFR2 angiogenesis marker (right). **m**, Inducible FOXF2 expression under the TetON promoter in human iPSCs, differentiation into iECs and Dx treatment for ChIP–seq experiments. Panels **a**, **g** and **m** were created with BioRender.com. **n**, Validation of FOXF2 expression in iECs with qPCR (left), ICC for mCherry expression marker (middle; scale bar, 20 μm) and western blotting (right). **o**, Top transcription factor motifs in FOXF2 peaks. **p**, Enrichment analysis of biological processes of *FOXF2* target genes with a minimum of ten observed binding sites and that further showed a significant enrichment compared to the values expected from a Poisson model. **q**, Genome browser screenshot of *TIE2*, *FOXO1*, *VEGFR2* and *VEGFR1* genes showing binding of FOXF2 (black), H3K27ac (red) and encode cREs overlapping with FOXF2 binding sites. **r**, Suggested mechanism by which Foxf2 maintains BEC function—Foxf2-mediated activation of Tie2 and VegfR signaling pathways inducing Pi3k and Akt phosphorylation (top); pAkt-driven activation of Nos3 inducing NO production (bottom-left); pAkt-induced phosphorylation and nuclear exclusion of Foxo1 (bottom-right); nuclear unphosphorylated Foxo1 regulates the transcription of Angpt2 and Nos3. Data are presented as mean ± s.d., comparison by two-tailed unpaired *t* test, ***P < 0.001; **P < 0.01; *P < 0.05 (**d**, **e**, **j**–**l** and **n**). *n* = 6 mice per group, pooled into *n* = 3 samples per condition (**b** and **c**). *n* = 4 mice per group (**d**). Foxf2, Foxq1 and Foxo1, *n* = 6 mice per group; Foxc1, *n* = 5 Ctrl and *n* = 4 iECKO mice per group (**e**). *n* = 4 mice per group (**f**). *n* = 5 WT and *n* = 6 KO iEC samples per group (**h**, **i** and **j**). *n* = 4 iEC samples per group (**k**, **l** and **n**). The number of iEC samples reflects technical replicates (**j**–**l**). ip, intraperitoneal injection; aECs, arterial endothelial cells; vECs, venous endothelial cells; capECs, capillary endothelial cells; FE, count, number of significantly altered proteins; #, position of GO term based on FE; one-tailed Fisher’s exact test, *P* < 0.05 (**b**, **h** and **p**); the exact *P* values are presented in source data file; RHP, retrohippocampal region; Dx, doxycyclin; cREs, *cis*-regulatory elements. Source data

**Extended Data Fig. 1:**
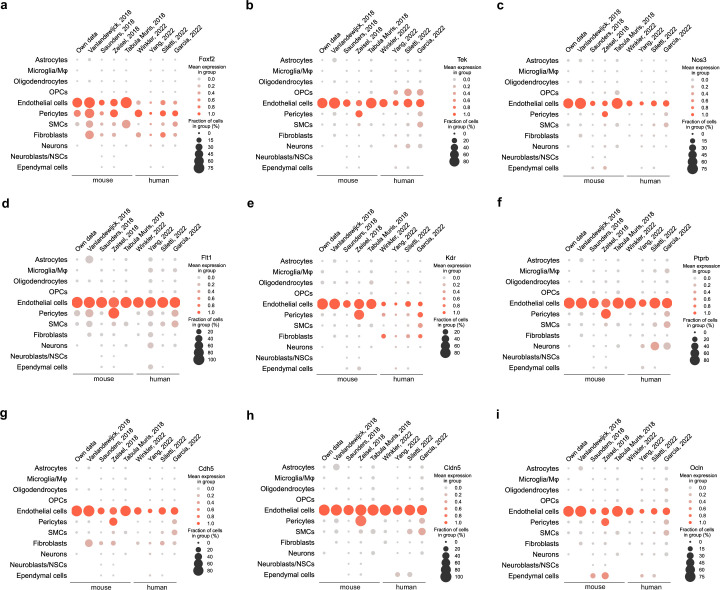
Comparative analysis of selected genes in different brain cell types based on single-cell RNA sequencing data (own results and published data). Expression of Foxf2 (**a**), Tie2 (**b**), Nos3 (**c**), Vegfr1 (Flt1) (**d**), Vegfr2 (Kdr) (**e**), Ptprb (**f**) and cell junction genes (**g**– **i**). Data are based on a total of 4.4 million cells (mean log-normalized expression values are scaled to (0,1) within each dataset by min-max normalization).

Given the causal role of Foxf2 in SVD and stroke and the involvement of vascular, glial and neuronal cells in mediating disease manifestations, we sought to obtain an overview of Foxf2 expression in adult mice. To this end, we performed single-cell RNA sequencing (scRNA-seq) on brains from 6-month-old mice and compiled the results with previously published scRNA-seq data from mouse^26,41–43^ and human brain^44–47^. Foxf2 is predominantly expressed in BECs and pericytes and largely absent in glia and neurons (Extended Data Fig. 1a). To investigate the role of Foxf2 in the maintenance of BEC function and the mechanisms linking Foxf2 to stroke and SVD, we generated mice with inducible deletion of Foxf2 in ECs (Cdh5-Cre^ERT2^;Foxf2^fl/fl^, hereafter Foxf2^iECKO^; Fig. 1a). Foxf2^fl/fl^ littermates were used as control animals (Ctrl). Foxf2^iECKO^ and Ctrl mice received tamoxifen injections at 3 months and were analyzed at 6 months of age.

**Extended Data Fig. 2:**
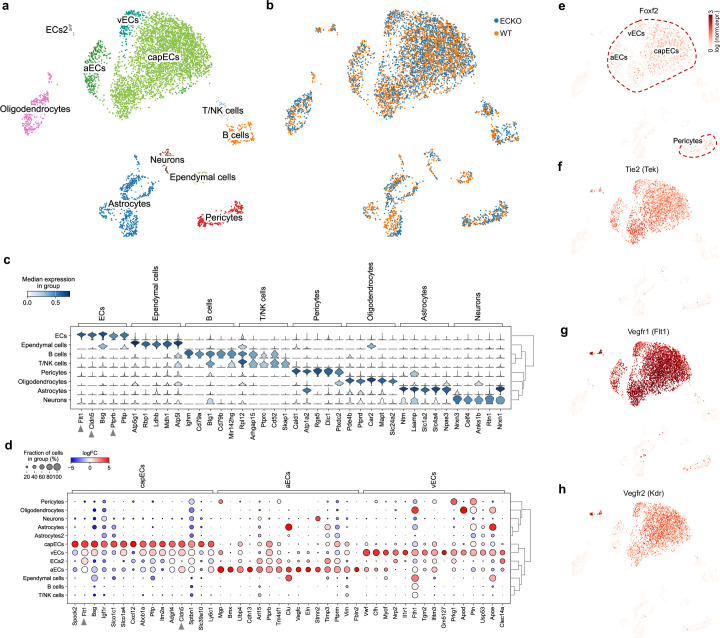
scRNA-seq analysis of brain endothelial cells from Foxf2^iECKO^ and Ctrl mice. **a**,**b**, UMAP embedding of BEC-enriched cell preparations from Foxf2^iECKO^ and Ctrl mice, colored by cell type (**a**) and by genotype (**b**). **c**, Top 5 enriched marker genes per cell type showing median log-normalized expression scaled to (0,1) for each gene by min-max normalization. Genes that are expressed in <20% of a given cell type were excluded. **d**, Top 15 marker genes of zonation-dependent BEC subtypes. Dot size indicates percentage of cells expressing the gene, color represents log fold change (FC) of given cell type versus all other cells. **e**–**h**, log-normalized expression levels of Foxf2, Tie2 (Tek), Vegfr1 (Flt1) and Vegfr2 (Kdr) (**a**–**h**. Differential expression of marker genes based on Wilcoxon rank-sum test, n = 6 mice per group).

We first performed RNA-seq on mouse BECs and human iECs to investigate the transcriptional effects of Foxf2. scRNA-seq on mouse BECs enriched from whole-brain tissue of Foxf2^iECKO^ and Ctrl mice returned the expected subpopulations of BECs (Fig. 1a, Extended Data Fig. 2 and Supplementary Table 1). Focusing on the top-enriched genes of different cell types, we found *Flt1*, *Cldn5* and *Ptprb* to be among the top-enriched endothelial genes in ECs compared to other cell types (Extended Data Fig. 2c). *Flt1* and *Cldn5* were further enriched in capillary ECs compared to other endothelial subtypes (Extended Data Fig. 2d). In Gene Ontology (GO), enrichment analyses that focus on significantly downregulated transcripts, ‘positive regulation of cell migration and proliferation’, ‘angiogenesis’ and ‘cell adhesion’ were among the most significant biological processes dysregulated in Foxf2^iECKO^ mice (Fig. 1b). Examining the angiogenesis-related and cell-adhesion-related transcripts, we found Efnb2, Nrp1, Tie2, Vegfr1 (Flt1) and Vegfr2 (Kdr), as well as Cldn5, Ctnnb1, Itga6, Itgb1 and Ocln, to be downregulated in BECs (Fig. 1c). The expression level of Nos3, which is downstream of both Tie2 and Vegf receptor signaling, was likewise downregulated (Supplementary Table 1). qPCR on full brain tissue confirmed the reduction of Tie2 and Nos3 mRNA levels (Fig. 1d). Focusing on FOX transcription factors, we found a downregulation of Foxf2, Foxc1 and Foxq1, while the mRNA level of Foxo1 was upregulated (Fig. 1e).

**Extended Data Fig. 3:**
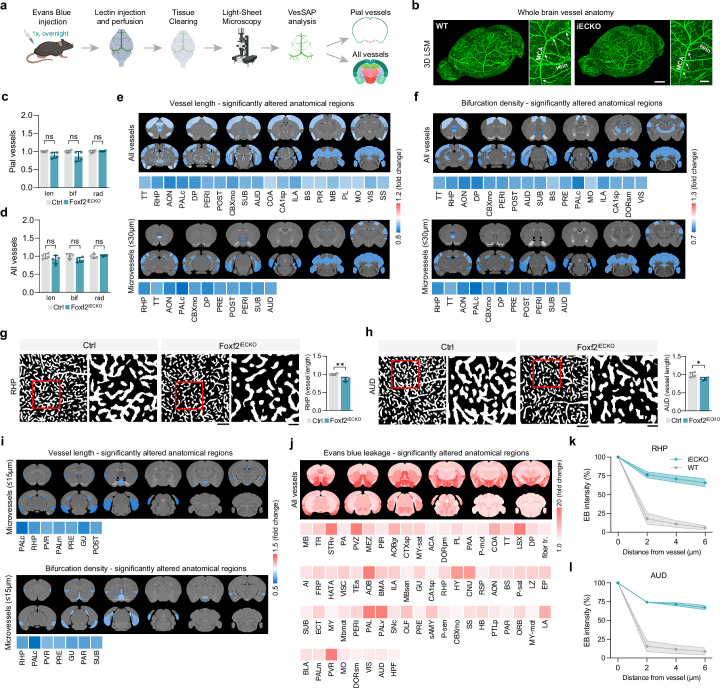
VesSAP analysis of whole brain vasculature and EB leakage of Foxf2^iECKO^ and Ctrl mice. **a**, Experimental outline. Mice with EC-specific Foxf2 deficiency were subjected to tissue clearing and light-sheet microscopy (LSM) followed by VesSAP analysis. Panel **a** was created with BioRender.com. **b**, 3D-LSM imaging of pial vasculature of Foxf2^iECKO^ and Ctrl mice (scale bar = 500 μm). High-magnification images show MCA collaterals (scale bar = 200 μm). **c**, Quantification of vessel metrics of pial vasculature. **d**, Quantification of vessel metrics of whole brain vasculature (**c**,**d**: len—vessel length, bif—bifurcation density, rad—vessel diameter). **e**, Anatomical regions showing a significantly altered vessel length in the overall dataset (top) and when focusing on microvessels (≤30 µm diameter) (bottom). **f**, Corresponding analyses for bifurcation density. **g**,**h**, Representative images and quantification of cortical regions with significantly reduced vessel density (scale bar = 100 μm, high-magnification images = 30 μm). **i**, Anatomical regions showing a significantly altered vessel length and bifurcation density, focusing on the smaller diameter range of microvessels (≤15 µm diameter). **j**, Whole brain mapping of EB leakage of Foxf2^iECKO^ versus Ctrl mice. Anatomical regions showing a significant EB leakage are highlighted in red. **k**,**l**, Quantification of distance-dependent intensity of EB along the brain vasculature in RHP and AUD anatomical region. **g** and **k**: retrohippocampal region, RHP; **h** and **l:** auditory cortex, AUD, **c**–**k**. n = 4 mice per group; comparison by two-tailed unpaired t-test, p < 0.05; **c**, **d** and **g**, **h**. Data are presented as mean ± s.d.

Given the observations on angiogenesis-related pathways, we then studied morphometric parameters of the brain vasculature using optical tissue clearing and light-sheet microscopy (LSM^48^; Extended Data Fig. 3a). Applying unsupervised VesSAP-based^49^ quantification, we found a reduction of vessel length and bifurcation density in several cortical regions of Foxf2^iECKO^ compared to Ctrl mice (Fig. 1f, Extended Data Fig. 3e–h and Supplementary Table 2). These differences were evident at the microvascular level—both in vessels with diameters ≤30 µm and ≤15 µm—whereas larger vessels (diameter, >30 µm) showed no significant difference between Foxf2^iECKO^ and Ctrl mice (Extended Data Fig. 3e,f,i and Supplementary Table 2). In contrast, the lengths, diameter and bifurcation density of pial vessels did not differ among genotypes (Extended Data Fig. 3b,c and Supplementary Table 2).

We then performed bulk RNA-seq in human endothelial cells (iECs) differentiated from FOXF2-deficient (hereafter FOXF2^KO^) and wild-type (hereafter WT) iPSC lines generated in parallel by CRISPR–Cas9 genome editing^50^ (Fig. 1g and Supplementary Table 3). Consistent with the results in mice, ‘cell adhesion’, ‘positive regulation of cell migration and proliferation’ and ‘angiogenesis’ were among the most affected biological processes in FOXF2^KO^ iECs in enrichment analyses of significantly downregulated transcripts (Fig. 1h). Focusing on angiogenesis receptors and cell-adhesion transcripts, we found VEGFR1, VEGFR2, EFNB2, ITGB1 and TGFR2, as well as CTNNB1, ITGA6 and ITGB1 to be downregulated (Fig. 1i). We further found a significant downregulation of TIE2 and NOS3 mRNA and protein levels in FOXF2^KO^ iECs using qPCR and western blotting, respectively, consistent with the results in Foxf2^iECKO^ mice (Fig. 1j,k). Given these observations and the results in mice, we performed proliferation assays in iECs and found a significantly lower proliferation rate in FOXF2^KO^ cells compared to WT cells (Fig. 1l, left). We further found a significant downregulation of VEGFR2 in FOXF2 deficiency (Fig. 1l, right).

**Extended Data Fig. 4:**
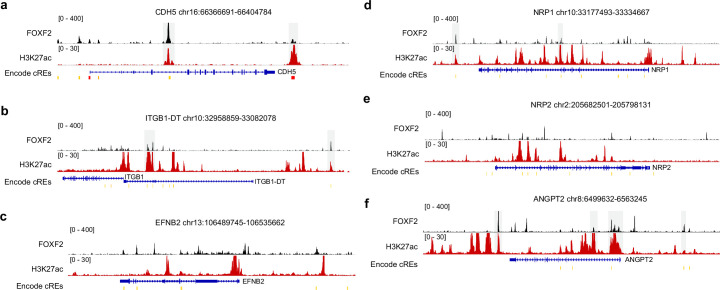
FOXF2 binding sites at selected target genes based on ChIP–sequencing. **a**–**f**, Genome browser screenshots of selected FOXF2 target genes showing FOXF2 (black), H3K27ac (red) and encode cREs overlapping with FOXF2 binding sites.

We then performed chromatin immunoprecipitation followed by sequencing (ChIP–seq) on human iPSC-derived ECs expressing epitope-tagged FOXF2–3xFLAG (Fig. 1m and Supplementary Table 4) to determine whether FOXF2 directly binds endothelial pathway genes. FOXF2–3xFLAG expression was confirmed by qPCR in combination with immunocytochemistry (ICC) and western blotting (Fig. 1n). In total, we identified 53,970 genomic binding sites associated with 13,444 genes. Sequence motif analysis identified the canonical Fox (Forkhead box) family motif and E-26 transformation-specific (ETS) family motif as the top hits present in 90.1% and 37.2% of the sites, respectively (Fig. 1o). For further analysis we focused on target genes with a minimum of ten observed binding sites, which also showed significant enrichment compared to expected values based on a Poisson model using two different strategies (Supplementary Table 4b,c). GO term enrichment analysis showed ‘angiogenesis’, ‘maintenance of BBB’ and ‘EC proliferation’ to be among the most strongly represented biological processes (Fig. 1p). Among the top FOXF2-bound genes were *TIE2* (TEK) and *VEGFR2* (KDR) with 3.35-fold and 10.08-fold enrichment (FE), respectively, suggesting a direct role of FOXF2 in activating TIE2 and VEGFR signaling (Fig. 1q). FOXF2 further bound to other target genes involved in angiogenesis (VEGFR1, EFNB2, NRP1) and cell adhesion (CDH5, ITGB1; Fig. 1q, Supplementary Table 4 and Extended Data Fig. 4) that were significantly downregulated in BECs from Foxf2^iECKO^ mice or FOXF2^KO^ human iECs in our RNA-seq experiments (Supplementary Tables 1 and 3). Interestingly, we further found FOXO1, which was significantly upregulated at mRNA level in Foxf2^iECKO^ mice (Supplementary Table 1) to be among the target gene candidates (Fig. 1q and Supplementary Table 4). Collectively, these findings indicate that FOXF2 acts as a transcriptional activator of Tie2 and other endothelial lineage-specific signaling genes (Fig. 1r).

### Endothelial Foxf2 deficiency causes BBB leakage and attenuates Tie2 signaling

**Fig. 2:**
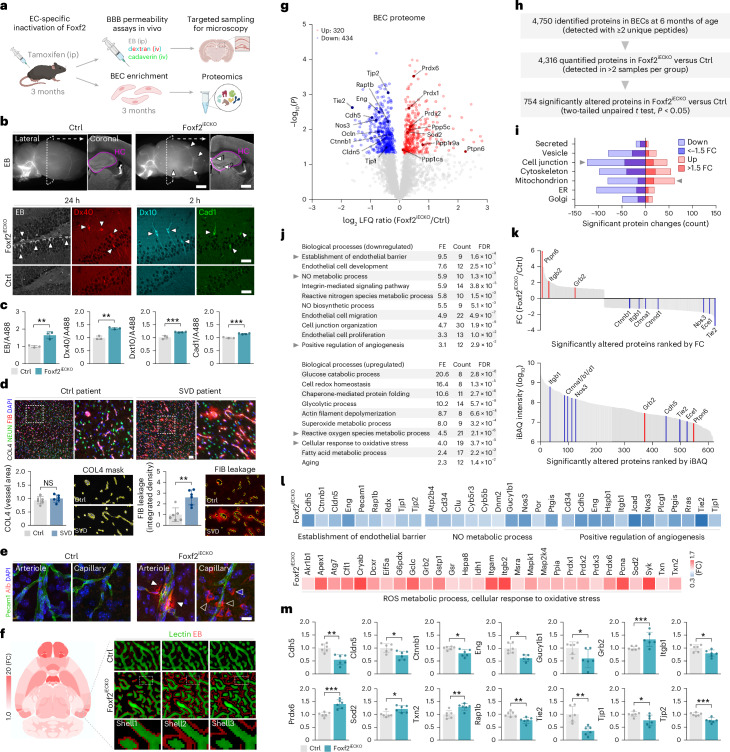
Endothelial Foxf2 deficiency causes BBB leakage and attenuates Tie2 signaling. **a**, Experimental outline. Mice with EC-specific inactivation of Foxf2 (Foxf2^iECKO^) at 3 months were assessed for BBB integrity and BEC proteome. iv, intravenous. Panel **a** was created with BioRender.com. **b**, Confocal microscopy images of tracer extravasation (EB, 65 kDa; TMR-conjugated dextran, 40 kDa; CB-dextran, 10 kDa; A555-cadaverine, 1 kDa) in Foxf2^iECKO^ versus Ctrl mice. Arrowheads indicate tracer extravasation (top) and cellular uptake (bottom). Scale bars, 2 mm and 0.5 mm (top), and 20 μm (bottom). HC, hippocampus. **c**, Quantification of tracer extravasation using fluorometry. The fluorescence intensities of all tracers were normalized to the autofluorescence signal of A488. **d**, Quantification of vessel density and fibrinogen (FIB) extravasation in histopathological sections from SVD patients and Ctrls (comparison by two-tailed unpaired *t* test, **P < 0.01; *n* = 6 patients per group; scale bar, 20 μm). **e**, Confocal microscopy images of Alb extravasation at the level of capillaries and arterioles, along with cellular uptake (scale bar, 10 μm). **f**, Whole-brain mapping of EB leakage of Foxf2^iECKO^ versus Ctrl mice. Brain regions with significant EB leakage are highlighted in red (left). LSM images depict the distance-dependent intensity of EB along the brain vasculature, categorized into three concentric shells (right). **g**, Volcano plot of log_2_ LFQ ratios (Foxf2^iECKO^ versus Ctrl) and −log_10_(*P*) of all quantified proteins from 6-month-old mice. Red and blue circles indicate proteins that were significantly upregulated and downregulated, respectively. Proteins marked with their corresponding gene names are associated with significantly enriched GO terms. **h**, Summary of LC–MS/MS and LFQ results. **i**, Subcellular localization of significantly dysregulated proteins. **j**, Enrichment analysis of biological processes of significantly dysregulated proteins in Foxf2^iECKO^ versus Ctrl mice based on the GO terms (FE; count, number of significantly altered proteins; FDR, adjusted *P* value of significantly enriched terms, *P* < 0.05). **k**, FC and iBAQ intensity ranking of significantly altered proteins in Foxf2^iECKO^ versus Ctrl mice. Red and blue lines indicate significantly upregulated and downregulated proteins, respectively, that are related to the Tie2-signaling pathway. **l**,**m**, Abundance of significantly downregulated proteins according to top-enriched Tie2-regulated biological processes (**l**, top, and **m**), and of significantly upregulated proteins related to ROS metabolic process and cellular response to oxidative stress (**l**, bottom). Comparison by two-tailed unpaired *t* test, *P* < 0.05 (**c**–**g**, **l** and **m**). Data are presented as mean ± s.d., ***P < 0.001; **P < 0.01; *P < 0.05 (**c**, **d** and **m**). *n* = 3 Ctrl-, EB and Dxt40, *n* = 3 iECKO-, Dxt10 and Cad1, *n* = 4 iECKO mice per group (**c**). *n* = 4 mice per group (**e**). *n* = 6 mice per group (**f**, **l** and **m**). The exact *P* values are presented in source data file. FC, fold change; iBAQ, intensity-based absolute quantification. Source data

To further characterize the phenotype of Foxf2^iECKO^ mice, we assessed BBB integrity using exogenous tracer injections (Fig. 2a) and found extravasation of Evans blue (EB; 65 kDa) and tetramethylrhodamine (TMR)-conjugated dextran (40 kDa) 24 h after dye injection in Foxf2^iECKO^ compared to Ctrl mice. We further detected extravasation of Cascade Blue (CB)-conjucated dextran (10 kDa) and Alexa Fluor 555 (A555)-conjucated cadaverine (1 kDa) 2 h after dye injection using confocal microscopy (Fig. 2b) and fluorometry (Fig. 2c). Thus, BBB leakage was also seen on histopathological sections from patients with pathologically confirmed SVD obtained through the Netherlands Brain Bank and processed in parallel with sections from control patients obtained through the same source. Specifically, we observed a significant increase in the extravasation of fibrinogen, as evidenced by the costaining of cortical microvessels with collagen type IV (COL4). In contrast, there was no difference in the density of cortical microvessels in SVD patients compared to controls (Fig. 2d and Supplementary Table 5).

Histological analysis of Foxf2^iECKO^ mice showed tracer uptake by parenchymal cells, particularly hippocampal neurons, consistent with findings in other mouse lines with BBB leakage^51,52^. In contrast, no tracer uptake was observed in Ctrl mice (Fig. 2b). Immunostaining for albumin (Alb) and Pecam1 revealed Alb extravasation at the level of both capillaries and arterioles, along with cellular Alb uptake consistent with the results obtained upon tracer injection (Fig. 2e).

**Extended Data Fig. 5:**
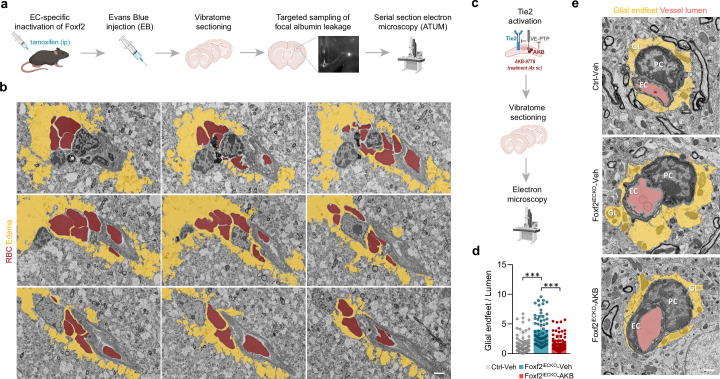
Cortical microhemorrhage in Foxf2^iECKO^ mice within a brain region showing focal albumin extravasation. **a**, Experimental outline. Mice with EC-specific inactivation of Foxf2 (Foxf2^iECKO^) at 3 months were injected with Evans blue intraperitoneally, followed by targeted sampling for focal albumin leakage and serial section electron microscopy (ATUM). **b**, Serial images of a cortical microhaemorrhage in Foxf2^iECKO^ mice within a brain region showing focal albumin extravasation. **c**, Experimental outline. Six-month-old Foxf2^iECKO^ mice were treated subcutaneously with AKB-9778 (AKB) or vehicle, followed by vibratome sectioning for scanning electron microscopy (SEM). Panels **a** and **c** were created with BioRender.com. **d**,**e**, Quantification and representative SEM images in vehicle-treated Ctrl, vehicle-treated Foxf2^iECKO^, and AKB-treated Foxf2^iECKO^ mice. **b**, Experiment was repeated three times; **d**, comparison by two-tailed unpaired t-test, ***p < 0.001, n = 4 mice per group, data are presented as mean ± s.d.; **b** and **e**, scale bar = 1 μm.

To quantify the degree of BBB leakage across the entire mouse brain, we developed a new bioinformatic approach based on our VesSAP pipeline^49^, registering EB leakage to Allen brain atlas regions. The results demonstrate widespread EB leakage within brain parenchyma of Foxf2^iECKO^ versus Ctrl mice (Fig. 2f and Extended Data Fig. 3j,k). Notably, the pattern of BBB leakage differed from the pattern of vessel density reduction (Extended Data Fig. 3i,j), suggesting distinct underlying mechanisms. Targeted examination of brain regions with BBB leakage further revealed occasional microhemorrhages in FOXF2^iECKO^ mice (Extended Data Fig. 5a,b) similar to mice with global inactivation of Foxf2 in adulthood^27^. Collectively, these findings suggest a requirement for Foxf2 expression in BECs for maintaining BBB integrity.

To identify the molecular and cellular pathways mediating the effects of Foxf2 in BECs, we then performed proteomic analysis of BECs. For this, we applied our previously published BEC enrichment protocol using magnetic-activated cell sorting (MACS) combined with liquid chromatography–mass spectrometry (LC–MS/MS)-based proteomics^53^ to 6-month-old animals (Fig. 2a). Proteomic analysis of isolated BECs captured a total of 4,750 proteins. Of these 4,750 proteins, 320 and 434 proteins were significantly upregulated and downregulated, respectively, in Foxf2^iECKO^ versus Ctrl mice (Fig. 2g,h and Supplementary Table 6).

In GO enrichment analyses of significantly downregulated proteins, ‘cell junction’ was the most abundant subcellular localization term (Fig. 2i). Focusing on biological processes, we found ‘establishment of endothelial barrier’, ‘NO metabolic process’ and ‘positive regulation of angiogenesis’ to be among the most affected categories (Fig. 2j). In contrast, ‘mitochondria’, ‘superoxide metabolic process’ and ‘aging’ were among the top GO terms based on the upregulated proteins (Fig. 2i,j; ordered based on FE and *P* values based on false discovery rate (FDR) of DAVID enrichment analysis). Fold-change ranking of significantly altered proteins marked Tie2 as one of the most strongly downregulated proteins, which further showed low abundance in intensity-based absolute quantification (iBAQ) analysis (Fig. 2k). Notably, several proteins involved in Tie2-regulated processes, including Nos3 and Ptgis (implicated in NO metabolic process), Rap1b, Tjp1, Cldn5 and Cdh5 (implicated in establishment of endothelial barrier) and Tie2, Eng and Itgb1 (implicated in angiogenesis), were downregulated in BECs from Foxf2^iECKO^ mice (Fig. 2l,m). In contrast, proteins involved in reactive oxygen species (ROS) metabolic process and cellular response to oxidative stress, including Prdx1–Prdx3, Prdx6 and Sod2, were upregulated. Collectively, these findings demonstrate a critical role of endothelial Foxf2 in maintaining BBB integrity and Tie2 signaling.

### Endothelial Foxf2 facilitates functional hyperemia and limits infarct size in adult mice through Tie2 signaling

**Fig. 3:**
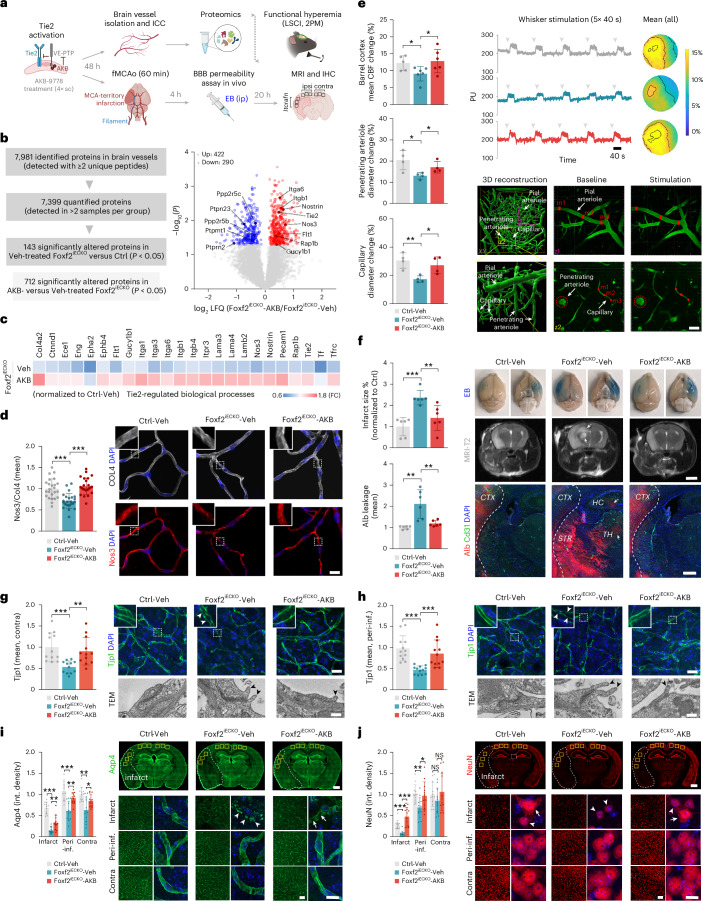
Endothelial Foxf2 facilitates functional hyperemia and limits infarct size in adult mice via Tie2 signaling. **a**, Experimental outline. Six-month-old Foxf2^iECKO^ mice were treated subcutaneously with the Tie2-activator AKB-9778 (AKB) followed by targeted assessments as indicated. Panel **a** was created with BioRender.com. **b**, Summary of the LC–MS/MS and LFQ results. Volcano plot of log_2_ LFQ ratios and −log_10_(*P*) of all quantified proteins in isolated brain vessels from AKB-9778 (AKB) and Veh-treated Foxf2^iECKO^ mice. Red and blue circles indicate proteins that were significantly upregulated and downregulated, respectively. Proteins related to Tie2 signaling are marked with their gene names. **c**, Abundance of Tie2–Nos3 signaling-related proteins that were rescued by the treatment with AKB-9778. **d**, ICC and quantification of Nos3 labeling in isolated brain microvessels (scale bar, 20 μm). **e**, Top, quantification of mean CBF changes within Barrel cortex obtained by LSCI with individual registrations and averaged CBF heatmaps following whisker stimulation. Yellow and blue colors indicate high changes and low changes in cortical perfusion compared to baseline, respectively. Bottom, representative images and quantification of vessel diameter changes of penetrating arterioles and capillaries following whisker stimulation (scale bar, 20 μm). **f**, Quantification of infarct size and Alb leakage 24 h after stroke in mice treated with either vehicle or AKB-9778 before fMCAO. Shown are exemplary images of the whole brain, MRI (scale bar, 5 mm), and confocal images (scale bar, 250 μm). **g**,**h**, Top right and left, immunohistochemistry and quantification of Tjp1 labeling in the contralateral cortex 24 h after stroke (**g**) and in the peri-infarct regions of the ipsilateral cortex (**h**). Scale bar, 20 μm (top right). White arrowheads indicate the loss of Tjp1 expression within tight junction regions. Bottom right, representative TEM image of endothelial tight junction regions from consecutive coronal sections (scale bar, 0.2 µm, bottom right). Black arrowheads indicate elongated tight junction protrusions. **i**,**j**, Quantification of glial endfeet (Aqp4) (**i**) and neurons (NeuN) (**j**) in the cortical regions of the infarct area, peri-infarct regions of the ipsilateral cortex and corresponding regions of the contralateral cortex 24 h after stroke. Scale bars, 1 mm (top), 60 μm (bottom left) and 10 μm (bottom right). Arrowheads indicate glial endfeet fragmentation (**i**) and neuronal death (**j**), whereas arrows indicate reduced Aqp4 density (**i**) and neuronal injury (**j**). Comparison by two-tailed unpaired *t* test, *P* < 0.05 (**b**–**j**). Data are presented as mean ± s.d., ***P < 0.001; **P < 0.01; *P < 0.05 (**d**–**j**). *n* = 4 mice per group (**b** and **c**). *n* = 24 images per group (**d**). CBF, *n* = 5 Ctrl-Veh-, *n* = 6 iECKO-Veh- and *n* = 6 iECKO-AKB mice per group; vessel diameter, *n* = 4 mice per group (**e**). *n* = 6 mice per group (**f**). *n* = 12 images per group (**g**,**h**, top, and **i**,**j**). Experiment was repeated thrice (**g**,**h**, bottom). The exact *P* values are presented in the source data file. sc, subcutaneous; MCA, middle cerebral artery; fMCAO, filamentous MCA occlusion; IHC, immunohistochemistry; ipsi, ipsilateral; contra, contralateral; peri-inf., peri-infarct area; Veh, vehicle; PU, perfusion unit; int., integrated; TEM, transmission electron microscopy. Source data

To further characterize the role of endothelial Foxf2 in maintaining vascular function and to examine the possible mediating effect of Tie2 signaling, we pharmacologically modulated Tie2 activity in mice and performed subsequent in vivo analyses, along with studies on isolated brain vessels. Specifically, we applied AKB-9778, a selective small-molecule inhibitor of vascular endothelial protein tyrosine phosphatase (VE-PTP, Ptprb), previously shown to stabilize the vasculature through Tie2 activation29,54 (Fig. 3a, top).

**Extended Data Fig. 6:**
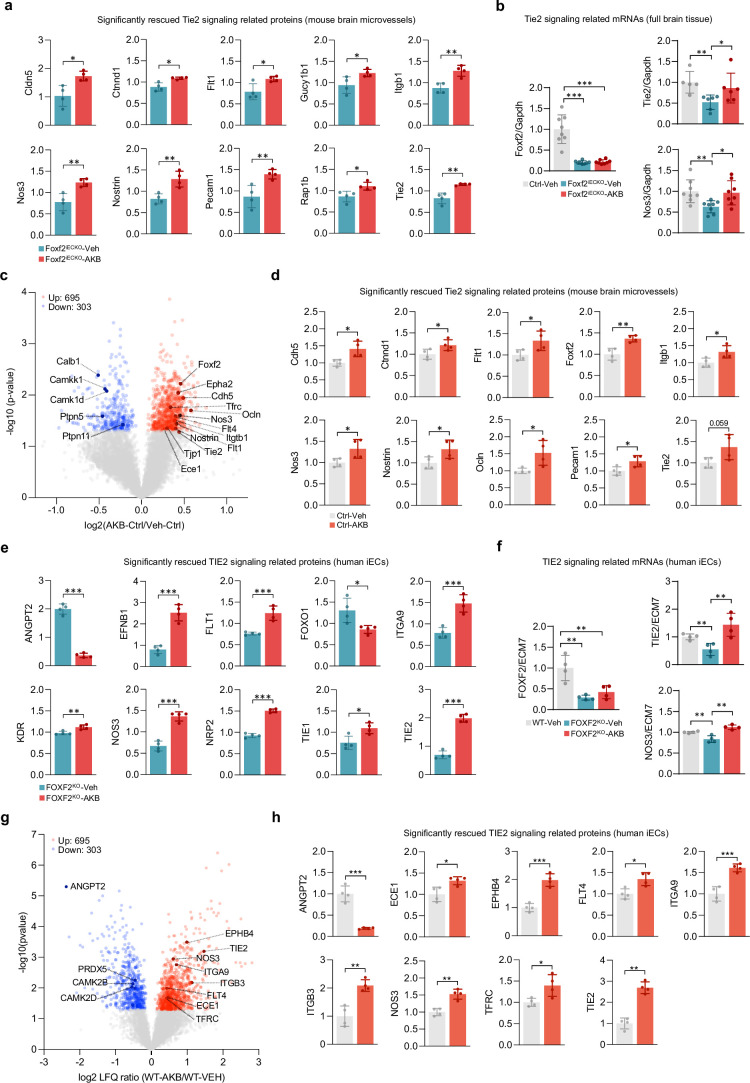
AKB-9778 rescues Tie2-signaling-related protein and mRNA changes in mouse brain microvessels and human iECs induced by Foxf2 deficiency. **a**, Abundance of Tie2-signaling-related proteins that were rescued in FOXF2^iECKO^ mice by treatment with AKB-9778. **b**, Relative mRNA abundance of selected transcripts in full brain tissue from vehicle (Veh) treated Ctrl mice and vehicle or AKB-9778 (AKB) treated FOXF2^iECKO^ mice (normalized to Gapdh and Ctrl). **c**, Volcano plot of all quantified proteins in isolated brain vessels from AKB-9778 (AKB) and vehicle (Veh) treated Ctrl mice. **d**, Abundance of selected Tie2-signaling-related proteins that were increased in Ctrl mice by treatment with AKB-9778. **e**, Abundance of TIE2-signaling-related significantly altered and rescued proteins upon AKB-9778 treatment. **f**, Relative mRNA abundance of selected transcripts in vehicle-treated human WT iECs and vehicle or AKB-9778 (AKB) treated FOXF2^KO^ iECs (normalized to GAPDH and WT). **g**, Volcano plot of all quantified proteins in isolated brain vessels from AKB-9778 (AKB) and vehicle (Veh) treated WT iECs. **h**, Abundance of selected TIE2-signaling-related proteins that were increased in WT iECs by treatment with AKB-9778. **a**–**h**, Comparison by two-tailed unpaired t-test, p < 0.05; **a**,**b**, **d**–**f**, **h**, Data are presented as mean ± s.d.; ***p < 0.001; **p < 0.01; *p < 0.05; **a**, **c**,**d**, n = 4 mice per group; **b**, Foxf2 and Nos3: n = 8 mice per group, Tie2: n = 5 Ctrl-Veh, n = 6 iECKO-Veh, and n = 6 iECKO-AKB mice per group; **e**–**h**, n = 4 iEC samples per group (the number of iEC samples reflects technical replicates); **c**,**g**, Red and blue circles indicate proteins that were significantly upregulated and downregulated, respectively. Proteins related to Tie2 signaling are marked with their gene names.

Mass-spectrometry analysis of isolated brain vessels from vehicle-treated Foxf2^iECKO^ and Ctrl mice (Foxf2^iECKO^-Veh and Ctrl-Veh, respectively) confirmed the dysregulation of multiple proteins related to Tie2 signaling (Fig. 3b,c and Supplementary Table 7a). Treatment of Foxf2^iECKO^ mice with AKB-9778 for 48 h restored their levels (Fig. 3b,c and Extended Data Fig. 6a). Specifically, the abundance of Nos3, Nostrin and Gucy1b1 (involved in NO metabolic process), Cldn5, Ctnnd1, Pecam1 and Rap1b (involved in establishment of endothelial barrier) and Tie2, Flt1 and Itgb1 (involved in angiogenesis) was upregulated upon Tie2 activation (Fig. 3b,c and Extended Data Fig. 6a,b). Restoration of Nos3 was further confirmed by immunolabeling of isolated brain microvessels (Fig. 3d). The treatment of Ctrl mice with AKB-9778 induced similar changes, including increased abundance of Nos3, Tie2 and Flt1, although the effect sizes were notably smaller compared to Foxf2^iECKO^ mice (Extended Data Fig. 6c,d and Supplementary Table 7b). We further found that Foxf2 expression was upregulated (Extended Data Fig. 6c,d) upon AKB-9778 treatment, potentially accounting for the increased levels of Tie2 and other Foxf2 target genes identified in our ChIP–seq analyses (Fig. 1).

Given the involvement of Nos3 in the regulation of CBF^54–57^ and the known impairment of cerebrovascular reactivity in SVD^2,6,7,11,58,59^, we then explored the consequences of Foxf2 deficiency on functional hyperemia using laser speckle contrast imaging (LSCI) and two-photon microscopy (Fig. 3a, top, and Fig. 3e). Quantification of cerebral perfusion in Barrel cortex after whisker stimulation revealed a reduction of functional hyperemia in endothelial-specific FoxF2 deficient (vehicle-treated Foxf2^iECKO^) compared to Ctrl mice. Yet, induction of Tie2 signaling by AKB-9778 in Foxf2^iECKO^ mice efficiently restored functional hyperemia (Fig. 3e, top). Moreover, two-photon microscopy revealed a reduced dilation of capillaries and penetrating arterioles in Foxf2^iECKO^ mice after whisker stimulation that was restored by the treatment with the Tie2 activator (Fig. 3e, bottom).

**Extended Data Fig. 7:**
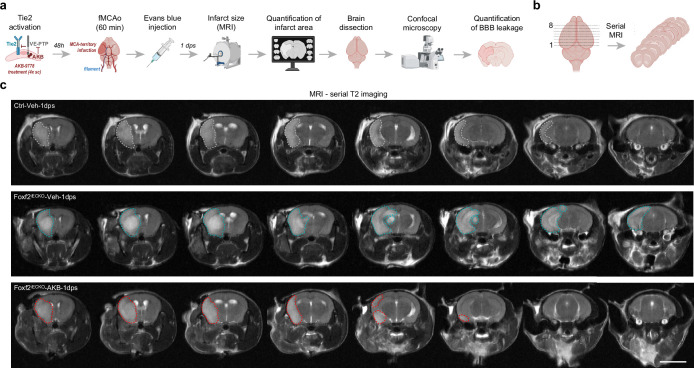
Assessment of infarct size and BBB leakage 1 day after stroke in Foxf2^iECKO^ and Ctrl mice treated with either vehicle or AKB-9778 prior to fMCAO. **a**, Experimental outline. Vehicle or AKB-9778-treated Foxf2^iECKO^ and Ctrl mice underwent middle cerebral artery occlusion (MCAO) followed by Evans blue injection and micro-MRI prior to brain dissection and confocal imaging. Infarct size was determined 1 day after experimental stroke induction (1 dps). **b**, Serial coronal MRI scans of about 8 consecutive regions. Panels **a** and **b** were created with BioRender.com. **c**, Exemplary magnetic resonance scans from Ctrl-Veh, Foxf2^iECKO^-Veh, and Foxf2^iECKO^-AKB mice. Shown are consecutive slices (T2-weighted sequence), scale bar = 5 mm.

To investigate the consequences of EC-specific Foxf2 deficiency on susceptibility to cerebral ischemia, we subjected mice to experimental stroke by transient middle cerebral artery occlusion (MCAO; Fig. 3a, bottom, and Extended Data Fig. 7a,b). Given our observations on BBB leakage in naive animals, we assessed both infarct size and BBB integrity at 24 h after stroke. Quantification of infarct size and BBB breakdown by magnetic resonance imaging (MRI) and confocal microscopy, respectively, revealed larger infarcts and more extensive Alb leakage in Foxf2^iECKO^ compared to Ctrl mice. The treatment of Foxf2^iECKO^ mice with AKB-9779 resulted in smaller infarct sizes and reduced Alb leakage compared with vehicle treatment (Fig. 3f and Extended Data Fig. 7c). As demonstrated by the three-dimensional (3D) vascular morphometry^49^ analyses, there was no difference in the lengths, diameters and bifurcation densities of pial vessels between genotypes arguing against differences in collateralization as a cause of differences in infarct sizes (Extended Data Fig. 3b,c and Supplementary Table 2).

**Extended Data Fig. 8:**
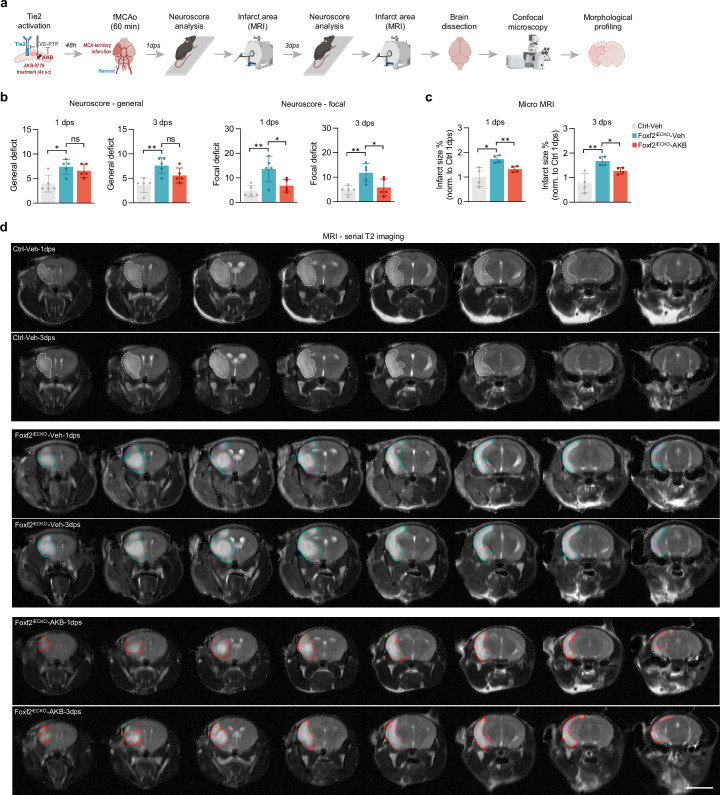
Assessment of neurological deficits and infarct size 1 and 3 days after stroke in Foxf2^iECKO^ and Ctrl mice treated with either vehicle or AKB-9778 prior to fMCAO. **a**, Experimental outline. Vehicle or AKB-9778-treated Foxf2^iECKO^ and Ctrl mice underwent middle cerebral artery occlusion (MCAO) followed by Neuroscore analysis and micro-MRI prior to brain dissection and confocal imaging. Neurological deficits and infarct size were determined 1 and 3 days after experimental stroke induction (1 and 3 days post-stroke, dps, respectively). Morphological profiling was performed 3 dps. Panel **a** was created with BioRender.com. **b**,**c**, Quantification of general and focal neurological deficit (**b**), and infarct size (**c**) 1 and 3 dps. **d**, Exemplary magnetic resonance scans from Ctrl-Veh, Foxf2^iECKO^-Veh, and Foxf2^iECKO^-AKB mice 1 and 3 dps. Shown are consecutive slices (T2-weighted sequence), scale bar = 5 mm. **b**,**c**, Data are presented as mean ± s.d.; comparison by two-tailed unpaired t-test, **p < 0.01; *p < 0.05; **b**, n = 5 mice per group, **c**. n = 4 mice per group.

**Extended Data Fig. 9:**
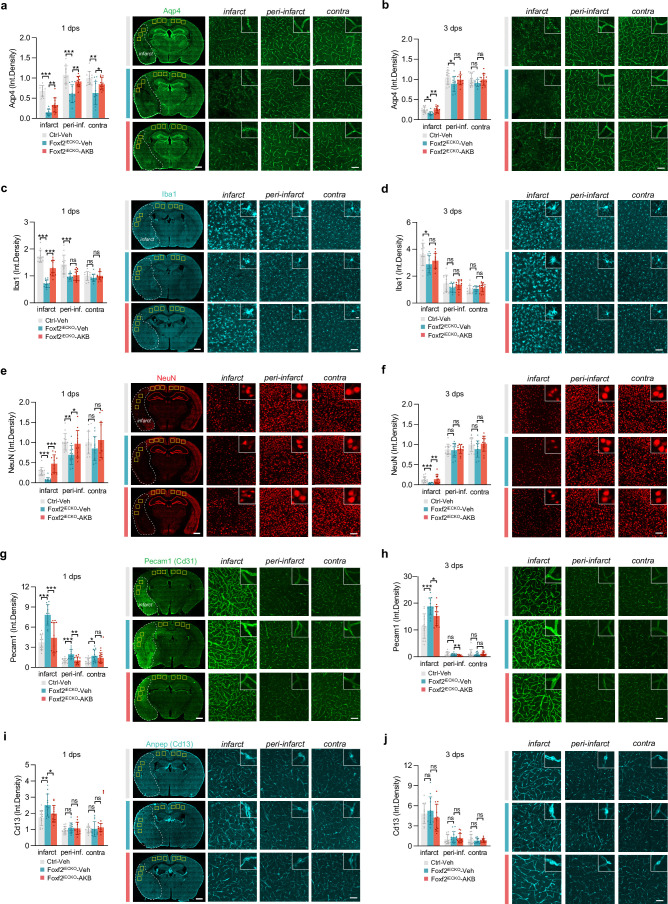
Morphological profiling of cortical regions in the infarct area, peri-infarct regions of the ipsilateral cortex, and corresponding regions of the contralateral cortex 1 and 3 days after stroke in Foxf2^iECKO^ and Ctrl mice treated with either vehicle or AKB-9778 prior to fMCAO. Quantification of the integrated density of glial endfeet (Aqp4) (**a**,**b**), microglia (Iba1) (**c**,**d**), neurons (NeuN) (**e**,**f**) and cell junctions (Cd31) (**g**,**h**). **i**,**f**, Pericytes (Cd13) of cortical regions in the infarct area, peri-infarct regions of the ipsilateral cortex, and corresponding regions of the contralateral cortex 1 and 3 days after experimental stroke induction (1 and 3 dps, respectively) (**a**–**j**, Data are presented as mean ± s.d.; comparison by two-tailed unpaired t-test, ***p < 0.001; **p < 0.01; *p < 0.05; n = 12 images per group; Scale bars = 60 μm).

**Extended Data Fig. 10:**
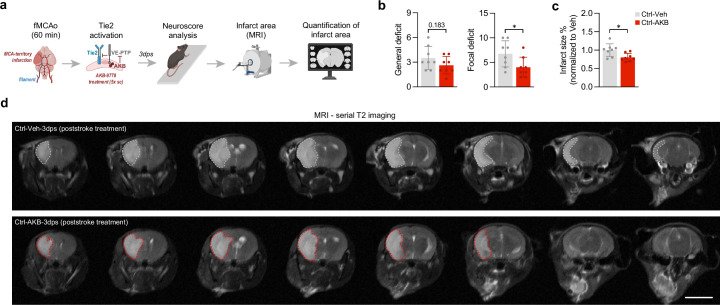
Assessment of neurological deficits and infarct size 3 days after stroke in control mice treated with either vehicle or AKB-9778 after fMCAO. **a**, Experimental outline. Ctrl mice underwent middle cerebral artery occlusion (MCAO) followed by vehicle or AKB-9778 treatment and micro-MRI. Neurological deficits and infarct size were determined 3 days after experimental stroke induction (3 dps). Panel **a** was created with BioRender.com. **b**,**c**, Quantification of general and focal neurological deficits (**b**), and infarct size (**c**) 3 dps in mice treated with either vehicle or AKB-9778 after fMCAO. **d**, Exemplary magnetic resonance scans from Ctrl-Veh, and Ctrl-AKB mice. Shown are consecutive slices (T2-weighted sequence), scale bar = 5 mm. **b**,**c**, Comparison by two-tailed unpaired t-test, *p < 0.05; n = 8 mice per group.

To further investigate the effects of Foxf2 deficiency on stroke outcome, we performed neuroscores, MRI and morphological profiling (cortical regions in the infarct area, peri-infarct areas of the ipsilateral cortex and corresponding regions of the contralateral cortex) during both the acute (1-day after stroke (1 dps)) and subacute (3 dps) phases (Extended Data Fig. 8a). Neuroscore analysis revealed more severe deficits in Foxf2^iECKO^ compared to Ctrl mice at both time points. Deficits of focal neurological function were restored by AKB-9778 treatment (Extended Data Fig. 8b). The genotype-dependent difference in infarct size persisted into the subacute phase (Extended Data Fig. 8c). Morphological analysis of tight junction regions in the contralateral cortex (Fig. 3g) and peri-infarct areas of the ipsilateral cortex (Fig. 3h) 24 h after stroke revealed reduced Tjp1 intensity and the presence of elongated endothelial protrusions in Foxf2^iECKO^-Veh compared to Ctrl-Veh mice, which were rescued by AKB treatment (Fig. 3g,h). Notably, Foxf2^iECKO^-Veh mice exhibited longer endothelial protrusions with basement membrane damage in the peri-infarct cortex compared to the corresponding contralateral areas, suggesting a possible mechanism for the more extensive BBB leakage in this region (Fig. 3g,h, bottom). Additional morphological analysis of glial endfeet, microglia, neurons, cell junctions and pericytes indicated that the differences among genotypes were most pronounced in the infarct area 24 h after stroke (Fig. 3i,j and Extended Data Fig. 9). Specifically, the integrated densities of Aqp4 (Extended Data Fig. 9a,b), Iba1 (Extended Data Fig. 9c,d) and NeuN (Extended Data Fig. 9e,f) were reduced, whereas Pecam1 (Extended Data Fig. 9g,h) and Cd13 (Extended Data Fig. 9i,j) were increased in Foxf2^iECKO^-Veh compared with Ctrl-Veh mice. High-resolution images further revealed fragmented glial endfeet in the infarct area of Foxf2^iECKO^ mice (Fig. 3g), possibly reflecting an exacerbation of the edematous glial endfeet observed in naive animals (Extended Data Fig. 5c–e). These alterations were all rescued by AKB treatment (Extended Data Figs. 5c–e and 9). Delayed initiation of AKB treatment—administered 2 h after stroke onset (5× ip injections in total)—significantly reduced both focal neurological deficits and infarct size in control mice (Extended Data Fig. 10), suggesting that AKB is also effective when administered after stroke onset. Collectively, these findings demonstrate that endothelial Foxf2 facilitates functional hyperemia and barrier integrity, resulting in a reduced ischemic lesion severity in adult mice through Tie2 signaling.

### AKB-9778 restores TIE2 signaling and NO production in human ECs lacking FOXF2

**Fig. 4:**
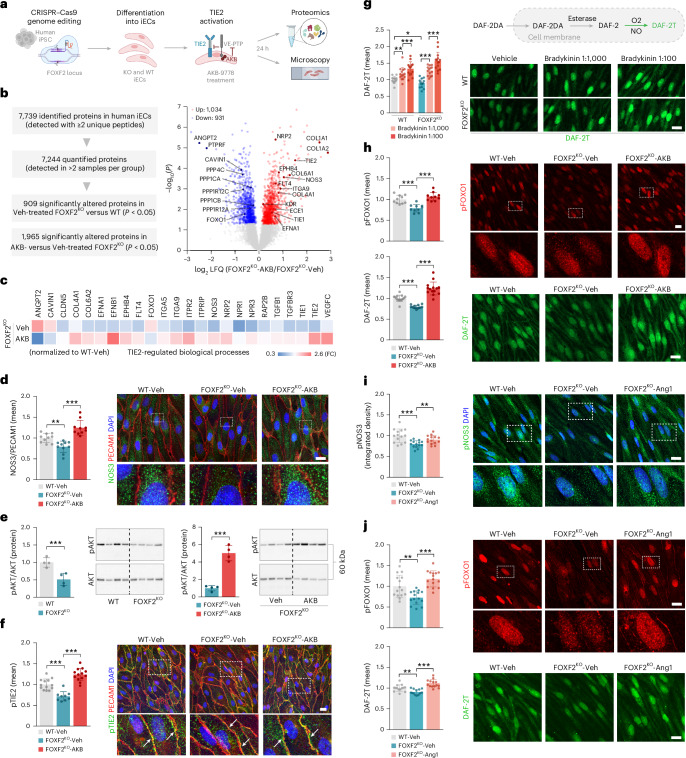
AKB-9778 restores TIE2 signaling and NO production in human ECs lacking FOXF2. **a**, Experimental outline. Human iPSCs were genome edited for FOXF2 deletion, differentiated into iECs, treated with AKB-9778 (AKB) or Veh, and subsequently subjected to proteomics and microscopic analysis. Panel **a** was created with BioRender.com. **b**, Summary of the LC–MS/MS and LFQ results. Volcano plot of log_2_ LFQ ratios (FOXF2^KO^-AKB versus FOXF2^KO^-Veh) and −log_10_(*P*) of all quantified proteins. Red and blue circles indicate proteins that were significantly upregulated and downregulated, respectively. Proteins related to TIE2 signaling are marked with their gene names. **c**, Abundance of TIE2-signaling-related proteins that were significantly altered in FOXF2^KO^-Veh versus WT-Veh cells (top, ‘Veh’) or rescued in FOXF2^KO^ cells upon AKB-9778 treatment (bottom, ‘AKB’). **d**, ICC and quantification of NOS3 in vehicle-treated WT, and vehicle-treated or AKB-treated FOXF2^KO^ iECs (scale bar, 20 μm). **e**, Western blot quantification of pAkt/Akt in untreated iECs and in vehicle-treated or AKB-treated iECs. **f**, ICC and quantification of pTIE2 in vehicle-treated or AKB-treated iECs (scale bar, 20 μm). Arrows mark membranous Tie2 staining. **g**, ICC and quantification of NO production in vehicle-treated or Bradykinin-treated iECs by DAF-2T fluorescence (scale bar, 20 μm). **h**, ICC and quantification of pFOXO1 (top) and NO production (bottom) in vehicle-treated or AKB-treated iECs (scale bar, 20 μm). **i**,**j**, ICC and quantification of pNOS3 (**i**), pFOXO1 (**j**, top) and NO production (**j**, bottom) in vehicle-treated or Ang1-treated iECs (scale bar, 20 μm). Comparison by two-tailed unpaired *t* test, *P* < 0.05 (**b**–**j**). Data are presented as mean ± s.d., ***P < 0.001; **P < 0.01; *P < 0.05 (**d**–**j**). *n* = 4 iEC samples per group (**b**, **c** and **e**). *n* = 10 images per group (**d** and **h**, top). *n* = 13 WT-Veh, *n* = 9 KO-Veh and *n* = 13 KO-AKB images (**f**). *n* = 14 images per group (**g**,**h**, bottom; **i**,**j**, bottom). *n* = 15 images per group; the exact *P* values are presented in the source data file. The number of iEC samples reflects technical replicates. Source data

To validate our results on Foxf2-related Tie2 signaling and explore their transferability to human cells, we studied human iPSC-derived endothelial cells (iECs)^50^. FOXF2^KO^ and WT cells were treated with AKB-9778 or vehicle and assessed by proteomics and microscopy (Fig. 4a). Similar to our results in mouse BECs (Fig. 2d), proteomic analysis revealed a dysregulation of multiple members of the TIE2-signaling pathway in vehicle-treated FOXF2^KO^ compared to WT iECs, including TIE2 and NOS3 (Figs. 1l and 4b,c and Supplementary Table 8). Moreover, the treatment of FOXF2^KO^ and WT cells with AKB-9778 restored the levels of multiple TIE2-signaling-related proteins (Fig. 4c, Extended Data Fig. 6e–h and Supplementary Table 8). Changes in the abundance of NOS3 protein were confirmed by ICC, further recapitulating the results in mice (Fig. 4d).

We then checked the abundance of phosphorylated AKT (pAKT) and found a significant downregulation in vehicle-treated FOXF2^KO^ compared to WT iECs (normalized to the levels of Akt), consistent with reduced TIE2 signaling and a restoration of pAkt levels by AKB-9778 treatment (Fig. 4e). Immunocytochemical analysis further showed a significant downregulation of both pTIE2 and pFOXO1 in vehicle-treated FOXF2^KO^ iECs and a rescue by AKB-9778 treatment (Fig. 4f,h, top). To investigate the functional effects of FOXF2 deficiency on NO metabolism and the potential to restore NO levels in FOXF2^KO^ cells, we pursued different pharmacological approaches and measured NO production using DAF-2T fluorescence (Fig. 4g, top). In all three approaches, NO production was reduced in vehicle-treated FOXF2^KO^ iECs compared to WT cells, consistent with our proteomics results (Fig. 4g,h,j, bottom). Treatment with Bradykinin, an enhancer of NO production^60^, increased NO levels in a concentration-dependent manner in both FOXF2^KO^ and WT cells (Fig. 4g). Treatment with AKB-9778 likewise increased NO levels in FOXF2^KO^ iECs (Fig. 4h, bottom). Given that AKB-9778 activates Tie2 signaling through VE-PTP inhibition, we further treated iECs with Ang1, the primary ligand that activates the Tie2 receptor^61^. Similar to AKB-9778, treatment with Ang1 rescued the reduced abundance of pFOXO1 and the decreased NO production in FOXF2^KO^ iECs, and further reduced the abundance of pNOS3 (Fig. 4i,j).

Collectively, these findings substantiate the role of Tie2 in mediating the effects of Foxf2 on endothelial dysfunction and demonstrate that AKB-9778 rescues the deficiency of TIE2 signaling and NO production in human iECs lacking FOXF2.

## Discussion

Stroke and SVD are associated with endothelial dysfunction^2,6,8,9,14^, but the underlying molecular pathways are insufficiently understood. Here we show that *Foxf2*, a major risk gene for stroke and SVD, acts as a transcriptional activator of Tie2 and other endothelial lineage-specific genes and maintains BEC function through Tie2 signaling. Specifically, we found that endothelial Foxf2 stabilizes the BBB both in naive animals and upon experimental stroke. We further demonstrate that endothelial Foxf2 promotes NO signaling, facilitates functional hyperemia and limits infarct size through Tie2 signaling. Pharmacological treatment with the Tie2-activator AKB-9778 restored the effects of Foxf2 deficiency on key outcomes, providing a therapeutic perspective.

The most striking phenotype in Foxf2^iECKO^ mice, besides a reduction in functional hyperemia and larger infarct sizes following experimental MCAO, was a loss of BBB integrity under both steady-state conditions and after cerebral ischemia. Several observations suggest that loss of Tie2 signaling is the predominant mechanism underlying BBB breakdown in Foxf2^iECKO^ mice. First, Tie2-regulated biological processes were among the most prominently downregulated proteins and pathways, respectively, in BECs from Foxf2^iECKO^ mice. Activation of Tie2 has been shown to stabilize EC junctions through the small GTPase Rap1 (refs. ^29,62^), the abundance of which was also reduced in Foxf2-deficient mice. Second, treatment with the Tie2-activator AKB-9778 almost completely rescued BBB leakage after experimental stroke. Third, the phenotype of Foxf2^iECKO^ mice mirrors that of previously reported adult mice with inducible overexpression of angiopoietin-2 (ANGPT2), a context-dependent antagonist of Ang1–Tie2 signaling^28,29,63^. Of note, Ang1 has a stimulating effect on Tie2, which protects the vasculature against barrier dysfunction^64,65^.

Further contributing to the observed loss of BBB integrity, we found a reduction in the abundance of tight and adherens junction proteins, including Tjp1 and Tjp2, Cldn5, Ocln, Cdh5 and Ctnnb1, and of integrins, including Itgb1 and Itga6, in BECs from Foxf2^iECKO^ mice. EC-restricted disruption of either Ctnnb1 (ref. ^66^) or Cldn5 (ref. ^67^) in adult mice has been shown to cause breakdown of the BBB. Itgb1 is essential for BBB integrity both under stable and vascular remodeling conditions^68,69^ and forms a coreceptor with Itga6 for BEC–extracellular matrix interactions^69,70^. Notably, AKB-9778 restored the abundance of Cdh5, Itg1b1 and Itga6 proteins in brain microvessels. Moreover, we identified several cell-adhesion-related genes as target genes of FOXF2 in our ChIP–seq analyses.

FOXF2 deficiency further results in an increased abundance of endothelial caveolae and enhanced caveolar transport^50^. The formation of endothelial caveolae and recruitment of transcellular pathways have been shown to account for the early phase of BBB breakdown in stroke^15^. Together with the impairment of paracellular pathways^15,71^, this could explain the exacerbation of infarct-related BBB breakdown in Foxf2^iECKO^ mice. Overall, these results establish a mechanistic link between *FOXF2*, a major risk gene for SVD and stroke, and BBB leakage.

Our finding of larger experimental infarct sizes in Foxf2^iECKO^ mice demonstrates a role of Foxf2 in stroke pathophysiology. The directionality of this effect is consistent with what would be expected from recent stroke GWAS, as risk alleles at FOXF2 are associated with lower FOXF2 expression^22^. Again, the most likely mechanism mediating the effect of endothelial Foxf2 deficiency on infarct size is the observed loss of Tie2 signaling, as treatment with AKB-9778 almost normalized infarct size to the level of Ctrl mice. Notably, adult mice with inducible overexpression of Angpt2 also develop larger experimental infarcts, which can be rescued by treatment with a Tie2 activator^63^. While AKB-9778 is a selective small-molecule inhibitor of VE-PTP known for stabilizing the vasculature through Tie2 activation^29,72^, its effects may not be entirely specific for the Tie2 receptor^73^ as VE-PTP also associates with Vegfr2 (refs. ^74,75^) and Cdh5 (refs. ^76,77^). In addition to larger infarct sizes, Foxf2^iECKO^ mice exhibited more severe neurological deficits and pronounced cellular alterations during both the acute and early subacute phases after stroke, all of which were rescued by AKB-9778 treatment. However, the effects of endothelial Foxf2 deficiency on stroke pathophysiology, particularly on angiogenesis during the later subacute and chronic phases, remain unexplored due to the high mortality rate in the Foxf2^fl/fl^;Cdh5-Cre line after MCAO.

A role of Tie2 signaling in stroke pathogenesis is further suggested by a recent GWAS that found an intronic variant in the *TIE2* (TEK) gene to be associated with risk of early onset stroke, although at subgenome threshold level^78^. Interestingly, recent GWAS also identified *NOS3* and *PRDM16*, a transcription factor regulating endothelial NO bioavailability^79^, as risk genes for ischemic stroke^18^. Endothelium-derived NO, the levels of which were reduced in our human ECs lacking FOXF2, is an important modulator of blood flow^32,33^ and mice lacking Nos3 exhibit larger cerebral infarcts after MCAO^80^ and BBB leakage^81^. Thus, Foxf2, Tie2 and Nos3 each have a protective role in the pathophysiology of stroke. Our results in mice and human iPSC-derived ECs establish a mechanistic link between FOXF2 and TIE2 signaling, with human data supporting its relevance to cerebrovascular disease in patients. Our finding of impaired functional hyperemia and rescue by AKB-9778 in Foxf2^iECKO^ mice further complements previous studies showing that interfering with endothelial pathways is a promising strategy to restore cerebrovascular function in SVD and stroke^6,7,11,82^.

Our ChIP–seq results indicate that FOXF2 directly regulates TIE2 transcription as a likely mechanism underlying the reduction of TIE2 mRNA levels and protein abundance in ECs with FOXF2 deficiency. This finding adds to previous work showing a transcriptional regulation of Tie2 by the synergistic action of Foxc1/Foxc2 and Etv2 (ref. ^83^). Interestingly, we found the expression levels of Foxc1 and Foxc2 to be downregulated in Foxf2^iECKO^ mice. Hence, Foxf2 regulates Tie2 transcription both directly and indirectly.

Our results imply a transcriptional effect of Foxf2 on several angiogenesis^28,84,85^ and cell-adhesion-related genes, including *VEGFR2*, *EFNB2* and *CDH5*, the expression levels of which were consistently reduced in mouse BECs and human iECs. Moreover, we found the levels of FOXO1, a major regulator of endothelial quiescence^34,35^ to be upregulated, consistent with the presence of multiple binding sites for FOXF2 in FOXO1. Our results in human iECs and in mice indeed suggest a possible effect of FOXF2 on EC proliferation and vascular remodeling, respectively. However, we did not address embryonic or early postnatal development, the critical period for angiogenesis^28,84,85^, as our focus was on maintaining EC function and exploring the possible role of FOXF2 in stroke and SVD. A global deficiency of Foxf2 leads to increased proliferation and impaired differentiation of pericytes during the embryonic phase. However, inactivation of Foxf2 in the adult phase results in no apparent difference in pericyte density^27^. Studies in mice with pericyte-specific inactivation of Foxf2 would be required to determine the specific contribution of Foxf2 expression in pericytes to SVD.

In conclusion, our findings demonstrate that *Foxf2*, a major risk gene for stroke and SVD, maintains EC function through Tie2 signaling. They further suggest that pharmacological targeting of EC-specific signaling pathways, as demonstrated here for the Tie2-activator AKB-9778, may limit disease manifestations. AKB-9778 has been tested in clinical trials for eye disease and has been shown to be well-tolerated^86,87^. Whether pharmacological activation of Tie2 signaling over an extended period of time has a favorable influence on neurovascular function warrants further investigation.

## Methods

### Animals

Animal experiments were performed in accordance with the German Animal Welfare Law (§4 TschG) and approved by the Government of Upper Bavaria (Vet_02-18-21). Mixed-sex groups with the same ratio of male and female mice were used for all experiments. Animals were maintained under standard conditions in a specific pathogen-free facility at 20–24 °C and 45–65% humidity on a 12-h light/12-h dark cycle, with access to food and water ad libitum.

Foxf2^fl/fl^;Cdh5-Cre (Foxf2^iECKO^) and Foxf2^fl/fl^ (Ctrl) mice were induced at 3 months of age using 3× intraperitoneal tamoxifen injection (0.25 mg g^−1^ body weight, dissolved in Miglyol 812)^27^. Experiments were performed at 6 months of age, which is 3 months after tamoxifen injection. Tissues were collected in parallel and on the same day. Proteomic, transcriptomic, immunohistochemical and in vivo analyses were done on four to eight mice per group (exact numbers are indicated in figure legends).

In all experiments, we assessed the level of tamoxifen-induced Foxf2 deficiency with qPCR from full brain tissue. Animals with <70% Foxf2 deletion efficiency were excluded from further analysis. In the in vivo experiments, the relative abundance of Foxf2 mRNA was assessed after the experiment was completed, resulting in the removal of *n* = 3 animals.

### Tissue collecting

Mice were deeply anesthetized using ketamine (100 mg kg^−1^, ip) -xylazine (10 mg kg^−1^, ip) and transcardially perfused with ice-cold 20 ml 1× Hank’s Balanced Salt Solution (HBSS). For BEC isolation, the dissected brains were kept in HBSS at 4 °C and immediately used for BEC preparation^53^. For vessel isolation, the dissected brains were frozen on dry ice and stored at −80 °C until preparation^88,89^. For immunohistochemical analysis, mice were transcardially perfused with HBSS and postfixed with 4% paraformaldehyde (PFA) overnight. The dissected brain samples were stored in HBSS at 4 °C until they were sectioned using a vibratome.

### BBB permeability assays

For the BBB permeability assays, EB (Sigma-Aldrich, E2129) was injected intraperitoneally, while A555-conjugated cadaverine (1 kDa; Invitrogen, A30677), CB-conjugated dextran (10 kDa; Invitrogen, D1976), and TMR-conjugated dextran (40 kDa; Invitrogen, D1845) were tail vein injected. EB and TMR-conjugated dextran were injected 24 h before animal perfusion. Thus, 555-conjugated cadaverine and CB-conjugated dextran were injected 2 h before perfusion using a second cohort. After HBSS perfusion, the right hemisphere was postfixed with 4% PFA overnight for confocal analysis.

### BEC isolation

BECs were isolated from the whole mouse brain as previously described^53^. In brief, whole mouse brains were placed on ice, minced with a scalpel and enzymatically digested using a modified version of the Adult Brain Dissociation kit (Miltenyi Biotec, 130-107-677). After tissue homogenization and filtration through 70-µm cell strainers (Corning, 431751), myelin and erythrocytes were removed using a 30% Percoll gradient (GE Healthcare, 17-5445-02) and Red Blood Cell Removal Solution (Miltenyi Biotec, 130-094-183), respectively. BECs were enriched from the single-cell suspension using CD31 MicroBeads (Miltenyi Biotec, 130-097-418) and MACS L-MACS buffer containing 0.25% BSA (BSA Fraction V; Sigma-Aldrich, 10735096001) and 2-mM EDTA (Thermo Fisher Scientific, 15575020) in PBS with calcium and magnesium (Corning, 21-030-CV). After CD31 enrichment, the cell suspension was washed twice with PBS and subsequently precipitated for further experiments.

### Brain vessel isolation

Brain vessels were isolated from the whole cerebrum as previously described^88,90^. In brief, cerebrum samples were placed on ice, minced with a scalpel and homogenized in 15 ml of cold minimal essential medium (Thermo Fisher Scientific, 11095080) using a glass tissue grinder (Wheaton). After dissociation, myelin was removed using a 15% Ficoll gradient, followed by resuspension of the pellet in PBS with 1% BSA (Fraction V; Sigma-Aldrich, 10735096001). Vessels were transferred to a 40-µm cell strainer (Corning, 431750) and extensively washed with cold PBS (with 250 ml). Isolated vessels were collected by washing the inverted cell strainer with PBS and then centrifuging at 3,000*g* for 5 min.

### Cell culture

Experiments on iPSCs were performed in accordance with relevant local guidelines and regulations. Work was done with the female iPSC line A18945 (Thermo Fisher Scientific, A18945; hPSCreg name TMOi001-A, RRID:CVCL_RM92). iPSCs were cultured and maintained on vitronectin-coated plates in Essential 8 Flex Medium (E8F; Thermo Fisher Scientific, A2858501) at 37 °C with 5% CO_2_ until reaching 80–85% confluency. iPSCs were passaged using PBS + 500-nM EDTA (Thermo Fisher Scientific, 15575020) and replated using E8F.

#### CRISPR–Cas9 genome editing

Genome editing of the FOXF2 locus was performed on the female iPSC line A18945, and the edited line was subsequently characterized in detail in ref. ^50^.

#### Differentiation of iPSC-derived endothelial cells (iECs)

iPSCs were differentiated into iECs and subsequently characterized^50^. In brief, cells were seeded onto gelatin-coated plates (Thermo Fisher Scientific, A1413302) at a density of 200 k cm^−^^2^ and mesoderm was induced for the next 5 days using Mesoderm Induction media (STEMCELL Technologies, 05220) for day 1–2 and APEL2 media (STEMCELL Technologies, 05270) for day 3–4. On day 5, iECs were positively selected by MACS using CDH magnetic beads (Miltenyi Biotec, 130-097-867) following the manufacturer’s instructions. iECs were further plated onto Collagen IV-coated plates (Sigma-Aldrich, C5533-5MG) in EC media (PromoCell, C-22011) supplemented with 50 ng ml^−1^ VEGF (Peprotech, 100-20). Cells were grown until they reached approximately 90% confluence and passaged with Trypsin-EDTA (Thermo Fisher Scientific, 25200056) up to five passages in a ratio of 1:2–1:6.

#### NO measurements

NO production was assessed using DAF-2DA compound (Enzo Life Sciences, ALX-620-056-M001). Cells were seeded onto Collagen IV-coated coverslips and cultured until they reached confluency. Cells were treated with 10-µM DAF-2DA diluted in phenol-free medium for 24 h. After incubation, cells were fixed with 4% PFA and mounted with Fluoromount medium (Sigma-Aldrich, F4680-25ML) for imaging.

#### Overexpression of FOXF2 in iPSCs

To overexpress FOXF2 in iECs, we integrated FOXF2 into a master cell line (MCL) containing a DOX-inducible cassette in the AAVS1 genomic safe harbor locus. For this purpose, a ‘landing pad’ containing FRT sites framing a GFP-resistance and hygromycin-resistance/thymidine kinase selection cassette was integrated into the AAVS1 site^91^ in the A18945 iPSC line. For the generation of the MCL, we used the pZ:F3-CAGGS GPHTK-F^91^ gene targeting vector (a gift from Catherine Verfaillie (Addgene plasmid 112666; http://n2t.net/addgene:112666; RRID:Addgene_112666)). Two million iPSCs were transfected with 32 μg of the gene targeting vector and 4 μg of AAVS1 locus-specific transcription activator-like effector nucleases (TALEN) plasmids, pTALEN-TD_hAAVS1-1L and pTALEN-TG_hAAVS1-1R in Ingenio electroporation solution (Mirus, MIR 50111) using the Gemini ×2 Electroporation System (BTX) with two pulses at 65 mV for 20 ms in a 1-mm cuvette (Thermo Fisher Scientific, 15437270). Cells expressing GFP-2A-HYG-TK were selected by sorting for GFP and with 50 μg ml^−1^ hygromycin B starting 3 days after electroporation. Single-cell colonies were picked and analyzed by genotyping PCR and qPCR, genome integrity was checked by standard trisomy 20 qPCR and molecular karyotyping (performed by Life&Brain GmbH), resulting in the selection of clone MCL-P1C11. Next, FOXF2–3xFLAG–mCherry was cut out from pPB[TetOn]–FOXF2–3xFLAG–mCherry using NotI/AleI (Vectorbuilder) and inserted into the vector pZ M2rtTA_CAGG TetON-Sox10 with GFP^92^ (a gift from Catherine Verfaillie (Addgene plasmid 115241; http://nst.net/addgene:115241; RRID:Addgene_115241)) by replacing Sox10-2a-GFP using AflII/MluI, so that FOXF2 can be expressed under a DOX-inducible promoter. A total of 14 µg of the purified FOXF2-containing plasmid was transfected together with 4 µg of a flippase-encoding plasmid (pCAG-Flpe-GFP, a gift from Connie Cepko; Addgene plasmid 13788; http://n2t.net/addgene:13788; RRID: Addgene_13788). Cells expressing FOXF2–3xFLAG–mCherry were selected by 350 ng ml^−1^ puromycin treatment 3 days after electroporation. Single-cell clone colonies were analyzed for the MCL generation. One selected clone was used for iEC differentiation and ChIP-seq experiments. Furthermore, 4 µg ml^−1^ doxycycline treatment for 48 h was used for FOXF2 overexpression.

### Protein extraction

#### Isolated mouse BECs and differentiated human iECs

Proteins were extracted from isolated mouse BECs and human iECs using RIPA buffer containing 150-mM NaCl (Roth, 3957.1), 1 M Tris–HCl pH 7.5 (Roth, 9090.3), 1% NP40 (Sigma-Aldrich, 74385), 0.5% deoxycholate (Roth, 3484.3), 0.1% SDS (Serva, 20765.03) and EDTA-free protease inhibitor cocktail (Roche, 4693159001). BEC and iEC samples were resuspended in 50 µl and 100 µl, respectively, and incubated on ice for 30 min followed by centrifugation at 18,000*g* for another 30 min at 4 °C. Supernatants were collected in protein-low-binding tubes and kept at −20 °C for further analysis.

#### Isolated vessels

Isolated vessels were lysed in a buffer containing 100 mM Tris–HCl, pH 7.6 (Roth, 9090.3), 4% SDS (Serva, 20765.03) and 100 mM DTT (Sigma-Aldrich, 3483-12-3) by homogenization with a dounce tissue grinder (Wheaton) followed by heating for 3 min at 95 °C. After lysis, samples were sonicated (30 s, amplitude 100%, duty cycle 50%) five times with intermediate cooling using VialTweeter sonicator (Hielscher). Samples were then centrifuged at 16,000*g* for 15 min at 4 °C. Supernatants were collected in protein-low-binding tubes and kept at −20 °C for further analysis.

### MS and data analysis

#### Sample preparation

The entire sample of acutely isolated BECs (~5 µg) or 20 µg of isolated brain vessel or full brain tissue lysates (as determined by the bicinchoninic acid (BCA) protein assay) was subjected to proteolytic digestion using the single-pot solid-phase enhanced sample preparation (SP3) method^93^. After 1:2 dilution with water, a benzonase digestion with 12.5 units was performed to remove remaining DNA/RNA. Proteins were reduced by the addition of dithiothreitol (Biozol) in 50 mM ammonium bicarbonate to a final concentration of 10 mM and incubated for 30 min at 37 °C. Cysteine residues were alkylated by adding iodoacetamide (Sigma-Aldrich) to a final concentration of 40 mM and incubating for 30 min at room temperature in the dark. Afterwards, the reaction was quenched by adding dithiothreitol.

Proteins were bound to 40 µg of a 1:1 mixture of hydrophilic and hydrophobic magnetic Sera-Mag SpeedBeads (GE Healthcare) using a final concentration of 70% (vol/vol) acetonitrile for 30 min at room temperature. Beads were washed four times with 200 µl of 80% (vol/vol) ethanol. For proteolytic digestion, LysC (Promega) was added in 20 µl of 50 mM ammonium bicarbonate with a protease-to-protein ratio of 1:80. Samples were incubated on a Thermomixer (Eppendorf) for 30 min at 1,000 rpm and 37 °C. Afterwards, trypsin (Promega) was added in 20 µl of 50 mM ammonium bicarbonate with a protease-to-protein ratio of 1:80, followed by an incubation for 16 h at room temperature. Beads were retained with a magnetic rack and the supernatants were collected. Next, 20 µl of 0.1% formic acid were added to the magnetic beads, followed by sonication for 30 s in a sonication bath (Hielscher Ultrasonics GmbH). The supernatants of each sample were combined, filtered through 0.22-µm spin filters (Costar Spin-X, Corning) to remove any remaining beads, and then dried by vacuum centrifugation. Dried peptides were dissolved in 20 µl of 0.1% formic acid. The peptide concentration after proteolytic digestion was estimated using the Qubit protein assay (Thermo Fisher Scientific).

#### MS

Acutely isolated BECs, brain microvessels and iPSC-derived human ECs were analyzed on a nanoElute nanoHPLC that was coupled to a TimsTOF pro mass spectrometer with a CaptiveSpray ion source (Bruker).

An amount of 350 ng of peptides was separated on in-house packed C18 analytical column (30 or 15 cm × 75 µm ID, ReproSil-Pur 120 C18-AQ, 1.9 µm, Maisch GmbH) using a binary gradient of water and acetonitrile (B) containing 0.1% formic acid at a flow rate of 300 nl min^−1^ and a column temperature of 50 °C. BECs (Fig. 1d) were separated with a 121-min gradient (0 min, 2% B; 2 min, 5% B; 92 min, 24% B; 112 min, 35% B; 121 min, 60% B). Isolated vessels and iPSC-derived human ECs were separated on a 15-cm column with a 90-min gradient (0 min, 2% B; 2 min, 5% B; 70 min, 24% B; 85 min, 35% B; 90 min, 60% B).

For BEC, a standard data-dependent acquisition parallel accumulation–serial fragmentation (DDA-PASEF) method with a cycle time of 1.1 s was used for spectrum acquisition. Briefly, ion accumulation and separation using trapped ion mobility spectrometry was set to a ramp time of 100 ms. One scan cycle included one trapped ion mobility spectrometry full MS scan and ten PASEF peptide fragmentation scans. The *m*/*z* scan range was set to 100–1,700 for both MS and MS/MS scans. The ion mobility scan range was set to 1/*k*_0_ 0.75–1.40. Isolated vessels and iPSC-derived human ECs were analyzed using DIA-PASEF. For isolated vessels, 26 windows with a width of 27 *m*/*z* and an overlap of 1 *m*/*z* covering a *m*/*z* range of 350–1,002. For iPSC-derived human ECs, 34 windows with a width of 26 *m*/*z* and an overlap of 1 *m*/*z* covering a *m*/*z* range of 350–1,201 were used. A ramp time of 100 ms, recording 2-*m*/*z* windows per PASEF scan, were applied for both DIA-PASEF methods.

#### Data analysis

The DDA-PASEF data were analyzed via MaxQuant software (maxquant.org, Max-Planck Institute Munich; v1.6.17)^94,95^. The MS data were searched against a canonical FASTA database of Mus musculus (one protein per gene, downloaded on 8 September 2020, comprising 21,997 entries) from UniProt. Trypsin was defined as protease. Two missed cleavages were allowed for the database search. The first search option was used to recalibrate the peptide masses within a 20 ppm window. For the main search, peptide mass tolerances were set to 10 ppm. Peptide fragment mass tolerances were set to 40 ppm. Carbamidomethylation of cysteine was defined as a static modification. Acetylation of the protein N terminal and oxidation of methionine were set as variable modifications. The false discovery rate (FDR) for both peptides and proteins was adjusted to less than 1%. Label-free quantification (LFQ) of proteins requires at least two ratio counts of unique peptides. The option ‘match between runs’ was enabled with a matching time of 0.7 min and an ion mobility window of 0.05 1/*k*_0_.

DIA-PASEF data were analyzed with the software DIA-NN (v1.8)^96^ to obtain protein LFQ intensities. Oxidation of methionines and acetylation of protein N terminal were defined as variable modifications, whereas carbamidomethylation of cysteines was defined as fixed modification. The precursor *m*/*z* ranges were limited from 350 to 1,001 for isolated vessels and 350–1,201 for iPSC-derived human ECs. The fragment ion *m*/*z* range was set to 200–1,700. Self-made spectral libraries with 11,456 protein groups and 131,497 precursors (FASTA database—canonical one protein per gene Mus musculus from UniProt, 25 January 2022, 21,994 entries) for isolated vessels and 116,40 protein groups and 138,112 precursors (FASTA database—canonical one protein per gene human protein from UniProt, 18 January 2022, 20,600 entries) for iPSC-derived human ECs were used. Peptide and peptide fragment tolerances were optimized by DIA-NN. The match between runs option was enabled. The protein LFQ intensities were log_2_ transformed and two-tailed unpaired *t* test was applied between the groups for statistical evaluation of differential protein abundance. Only proteins with at least three valid values per group were considered for relative quantification.

### Enrichment analysis

Enrichment analysis of biological processes (GOTERM_BP_DIRECT) of downregulated and upregulated proteins was performed using DAVID software^97,98^ (v2023q4) with Mus musculus as background dataset.

### Western blot and quantification

Protein lysates were analyzed by sodium dodecyl sulfate–polyacrylamide gel electrophoresis (SDS–PAGE) and transferred to 0.2-µm nitrocellulose membranes using the Mini-Protean and Trans-Blot system. After transfer, membranes were incubated in I-Block (Invitrogen, T2015) for 1 h at room temperature. Primary antibodies were incubated in the same buffer at 4 °C overnight and horseradish peroxidase-conjugated secondary antibodies for 1 h at room temperature. Detection was performed using chemiluminescence development (Immobilon ECL detection reagent, Merck Millipore) on a Fusion FX7 (Vilber Lourmat). Protein levels were quantified using ImageJ Gel analyzer (v1.52p).

### Immunolabeling

The details of primary and secondary antibodies for all experiments are specified in Supplementary Table 9.

#### Brain slices

Frozen human brain samples from *n* = 6 patients with sporadic cerebral SVD (mean age, 70.3 years) and *n* = 6 age-matched and sex-matched control participants without known cerebrovascular disease (mean age, 79.5 years) were obtained from the Netherlands Brain Bank (Netherlands Institute for Neuroscience; www.brainbank.nl). All material has been collected from donors for whom a written informed consent for a brain autopsy and the use of the material and clinical information for research purposes had been obtained. Brain samples were embedded in OCT for 10-µm-thick cryosectioning and fixed with ice-cold acetone for 10 min before immunostaining. PFA-fixed mouse brain samples were embedded in 3% agarose blocks for 100-µm-thick coronal vibratome sectioning.

Mouse free-floating and human cryosections were incubated in 3% BSA/Triton X-100 buffer for 1 h at room temperature for tissue permeabilization and blocking. Primary antibodies were diluted in 1% BSA/Triton X-100 buffer and incubated at 4 °C overnight, while secondary antibodies were diluted in PBS and incubated at room temperature for at least 2 h. After careful washing, DNA was stained using DAPI (Invitrogen, D1306; 1:2,000) at room temperature for 5 min. Brain slices were mounted using Fluoromount medium (Sigma-Aldrich, F4680-25ML).

#### Isolated vessels

After preparation vessels were immediately transferred onto microscope slides (Thermo Fisher Scientific, J1800AMNZ) and dried at room temperature. Next, vessels were fixed at −20 °C for 10 min using ice-cold 100% acetone. After fixation and washing, vessels were permeabilized and blocked with 3% BSA/PBS buffer. Primary antibodies were diluted in 1% BSA/PBS buffer and incubated at 4 °C overnight, while secondary antibodies were diluted in PBS and incubated at room temperature for 2 h. After washing, nuclei were stained with DAPI for 5 min at room temperature. Isolated vessels were then mounted using Fluoromount medium (Sigma-Aldrich, F4680-25ML).

#### Differentiated human endothelial cells (iECs)

Human iECs were seeded directly onto Collagen IV-coated coverslips and fixed using 4% PFA for 15 min at room temperature upon confluency. Cells were blocked using 1% BSA/PBS buffer for 1 h at room temperature. Primary antibodies were diluted in the same blocking buffer, while secondary antibodies were diluted in PBS. Primary antibodies were incubated overnight at 4 °C, while secondary antibodies were incubated at room temperature for at least 1 h. After washing, DNA was stained using DAPI and coverslips were mounted using Fluoromount medium (Sigma-Aldrich, F4680-25ML).

### Confocal microscopy and image analysis

Fluorescence images were acquired with Zeiss confocal microscope (LSM800 and LSN980) using ×10, ×40 and ×63 objectives. Images were processed and analyzed using ImageJ software (v1.52p).

### LSM and image analysis

For staining, imaging and analyzing the whole-brain pial vasculature of optically cleared intact mouse brains from Foxf2^iECKO^ and Ctrl mice, we followed our previously published VesSAP protocol^49^.

#### Vessel labeling and tissue preparation

For labeling the whole-brain vasculature, we first injected 150 μl (2% vol/vol% in saline) EB (Sigma-Aldrich, E2129) intraperitoneally into 6-month-old Foxf2^iECKO^ and Ctrl mice (*n* = 4 mice per group). Thus, 12 h after injection, we injected 0.25 mg wheat germ agglutinin conjugated to Alexa Fluor 594 dye (Thermo Fisher Scientific, W11262) in 150 μl PBS (pH 7.2) intravenously. Next, the fixed brains were optically cleared using the 3DISCO technique^48^.

#### Imaging of the cleared whole-brain samples with LSM

Imaging of the cleared whole-brain samples was performed using a ×4 objective lens (Olympus, XLFLUOR 340) equipped with an immersion corrected dipping cap mounted on a LaVision UltraII microscope coupled to a white light laser module (NKT SuperK Extreme EXW-12).

#### Reconstruction of the datasets from the tiling volumes

The TeraStitcher’s automatic global optimization function (v1.10.10) was used for 3D data reconstruction from the tiling volumes. To register our dataset to the reference atlas, we used the average template, the annotation file and the latest ontology of the current Allen mouse brain atlas CCFv3 201710.

#### Light-sheet data analysis

We used the vessel segmentation and analysis pipeline (VesSAP) to quantify the whole brain and pial vasculature to obtain the total vessel length, bifurcation density and average radius of vessels. All measures were then corrected by a constant to account for shrinkage due to fixation and clearing. Group comparison was done using two-tailed unpaired *t* test followed by a Tukey’s post hoc test. For the analysis of the pial vasculature, the registered brain atlas was iteratively eroded by 40 voxels along each of the three spatial dimensions. The resulting mask was then subtracted from the original brain atlas, producing a uniformly thick cortical surface region in which the vasculature was quantified. Microvessels were defined as vessels with one to three voxel radii (diameter: ≤30 µm) and one to two voxel radii (diameter: ≤15 µm). EB leakage analysis was done by generating 3D concentric shells around the vessel segmentation mask, with shell distances of one, two and three voxels, respectively (denoted as shells 1, 2 and 3). The mean intensity of the EB channel was computed for the voxels within each shell, excluding those with zero intensity values, and normalized to the mean intensity of the EB in the vascular mask in the major brain regions. The EB leakage of the major brain regions was averaged between hemispheres. Statistical group differences for each brain region were done with a two-tailed unpaired Student’s *t* test.

### Electron microscopy and image analysis

#### Scanning electron microscopy

For correlative analysis, mouse brain samples were perfusion fixed in 4% PFA, 2 mM calcium chloride in 1× PBS, pH 7.4 (Science Services). Coronal, 100-µm-thick vibratome sections were generated and every second section poststained for 24 h in EM fixative (4% PFA, 2.5% glutaraldehyde, 2 mM calcium chloride in 0.1 M cacodylate buffer). The remaining sections were stained for Alb and screened for BBB leakage by confocal microscopy. Adjacent sections to the ones selected by fluorescence microscopy were subjected to EM processing.

We applied a rOTO en bloc staining protocol including postfixation in 2% osmium tetroxide (EMS), 1.5% potassium ferricyanide (Sigma-Aldrich) in 0.1 M sodium cacodylate (Science Services) buffer (pH 7.4)^99^. Staining was enhanced by reaction with 1% thiocarbohydrazide (Sigma-Aldrich) for 45 min at 40 °C. The tissue was washed in water and incubated in 2% aqueous osmium tetroxide. It was then washed and further contrasted by overnight incubation in 1% aqueous uranyl acetate at 4 °C, followed by 2 h at 50 °C. Samples were dehydrated in an ascending ethanol series and infiltration with LX112 (LADD). Blocks were cured and trimmed (TRIM2; Leica).

#### Image analysis

Serial sections were taken with a 35° ultradiamond knife (Diatome) on an ATUMtome (Powertome, RMC) at a nominal cutting thickness of 100 nm and collected onto freshly plasma-treated (custom-built, based on Pelco easiGlow, adopted from M. Terasaki, University of Connecticut), carbon-coated Kapton tape (kindly provided by Jeff Lichtman and Richard Schalek). Tape stripes were assembled onto adhesive carbon tape (Science Services) attached to 4-inch silicon wafers (Siegert Wafer) and grounded by adhesive carbon tape strips (Science Services). EM micrographs were acquired on a Crossbeam Gemini 340 s.e.m. (Zeiss) as described. Hierarchical imaging of serial sections was performed by mapping the entire wafer at a 2,000-nm lateral resolution and acquiring entire tissue sections at medium resolution (100–200 nm). The region of interest was correlated by anatomical landmarks, including bleedings and vascular patterns and serial sections thereof acquired at 8 × 8 × 100 nm resolution. Serial section data were aligned by a sequence of automatic and manual processing steps in Fiji TrakEM2 (ref. ^100^).

### RNA extraction and cDNA synthesis

Total RNA from mouse brain cerebellum or human cell pellet was extracted using Trizol (Qiagen, 79306) and purified using the RNeasy mini kit (Qiagen, 74106) according to the manufacturer’s instructions. RNA concentration was determined using a NanoDrop spectrophotometer. RNA was stored at −80 °C until use. cDNA was synthesized from 250 ng to 1 µg RNA using the Omniscript RT kit (Qiagen, 205113) following the manufacturer’s instructions and stored at −20 °C.

### Quantitative real-time qPCR

SYBR Green master mix (Qiagen, 208056) was used to perform qPCR and detection was done in the Roche thermocycler. Primer sequences are listed in Supplementary Table 10.

### scRNA-seq of mouse and human brain

#### WT mouse brain scRNA-seq data analysis

Read processing was performed using 10x Genomics Cell Ranger (v6.0.0). After barcode assignment and UMI quantification, reads were aligned to the mouse reference genome mm10 (GENCODE vM23/Ensembl 98; 2020 A from 10x Genomics Cell Ranger). Further processing was performed using Scanpy (v1.9.1)^101^. Cells were excluded if they had ≤200 or ≥7,000 unique genes, or ≥20% mitochondrial gene counts. The count matrix was normalized (sc.pp.normalize_total) and log_(*x* + 1)_-transformed (sc.pp.log1p), before proceeding with dimensionality reduction and clustering (sc.tl.pca, sc.pp.neighbors with n_pcs=50, sc.tl.umap, sc.tl.leiden with a resolution of 1.1).

Cell types were annotated using known marker genes for ECs (Cldn5, Pecam1), pericytes (Vtn, Pdgfrb), smooth muscle cells (Acta2, Myocd), fibroblasts (Dcn, Col6a1, Col3a1), oligodendrocytes (Mbp, Enpp2), oligodendrocyte precursor cells (Cspg4, Pdgfra), neurons (Rbfox3, Tubb3), astrocytes (Aqp4, Aldoc), microglia (Aif1, Tmem119), monocytes/macrophages (Cd14, Itgb2, Cd86, Adgre1, Ccr2), other immune cells (Cd19, Cd3e, Il2rb, Lat, Ifng, S100a9) and ependymal cells (Pifo, Foxj1, Dynlrb2, Meig1). Cluster identities were manually verified using differential expression analysis based on Wilcoxon rank-sum tests (sc.tl.rank_genes_groups with method = ‘wilcoxon’). The expression of marker genes and the full analysis pipeline are available at github.com/simonmfr/foxf2-per-celltype/blob/manuscript/notebooks/sc_pp_ISD_2022.ipynb.

#### Comparative analysis of human and mouse brain scRNA-seq datasets

We compared our scRNA-seq data to eight independent single-cell/single-nucleus RNA-seq datasets from the mouse and human brain^26,41–47^. Each dataset was processed separately in Scanpy by first normalizing (sc.pp.normalize_total) and log-scaling (sc.pp.log1p) raw count matrices. Available cell annotations were verified using known marker genes (as described above) and then harmonized into major cell types (astrocytes, microglia/macrophages, oligodendrocytes, oligodendrocyte precursor cells, ECs, pericytes, smooth muscle cells, fibroblasts, neurons, neuroblasts/neural stem cells, ependymal cells). Cell types with <50 cells were excluded from the analysis. Next, we extracted mean scaled expression levels and the fraction of cells expressing the respective gene per cell type (sc.pl.dotplot). Overall, the analysis included 4,347,895 cells, of which 86,588 cells were annotated as ECs. Details of the analysis and the full code are available at https://github.com/simonmfr/foxf2-per-celltype/tree/manuscript.

### scRNA-seq of mouse BECs (Foxf2^iECKO^ versus WT)

#### BEC isolation

BECs were isolated from the whole mouse brain as previously described^53^ and sorted using fluorescence-activated cell sorting. After CD31 enrichment, cell suspension was resuspended in Flow Cytometry buffer (Invitrogen, 00-4222-26) and stained with CD11b (Invitrogen, 53-0112-82), CD45 (Invitrogen, 53-0451-82) and Fixable Viability Dye eFluor 780 (Invitrogen, 65-0865-14). Doublets and microglia population (defined as CD11b and CD45^+^) were gated out before fluorescence-activated cell sorting. Viable ECs were sorted into RPMI media (Invitrogen, 11835030) supplemented with 10% FBS.

#### Library preparation and sequencing

Fresh single-cell suspensions were centrifuged and resuspended in PBS containing 0.04% ultrapure BSA. Libraries for scRNA-seq were prepared using the Chromium Next GEM Single Cell 3′ Reagent Kits (v3.1; 10x Genomics Cell Ranger) following the manufacturer’s instructions. Libraries were sequenced on an Illumina HiSeq 4000.

#### Data analysis

Samples were preprocessed separately (two genotypes, each consisting of six mice). First, read processing was performed using 10x Genomics Cell Ranger (v7.1.0). After barcode assignment and UMI quantification, reads were aligned to the mouse reference genome mm10 (GENCODE vM23/Ensembl 98; 2020 A from 10x Genomics Cell Ranger). Further processing was performed using Scanpy (v1.9.6)^101^. Outlier cells were excluded if they had a median absolute deviation (MAD) ≥ 5 of the QC covariates log1p_total_counts, log1p_n_genes_by_counts and pct_counts_in_top_20_genes, or a MAD ≥ 3 for mitochondrial counts (pct_counts_mt)^102^. Next, background contamination (ambient RNA) was corrected using SoupX^103^, and genes not detected in at least 20 cells were filtered out. Doublet cells were detected using scDblFinder^104^ and excluded. Next, the count matrix was normalized (sc.pp.normalize_total) and log_(*x* + 1)_-transformed (sc.pp.log1p), before proceeding with dimensionality reduction and clustering (sc.tl.pca using 4,000 highly deviant genes^102^, sc.pp.neighbors with n_pcs=50, sc.tl.umap, sc.tl.leiden with a resolution of 1.5). Finally, all samples were integrated using Harmony^105^. Cell types and cell subtypes were iteratively annotated using known marker genes and a manual verification based on the differential expression analysis (as described in ‘WT mouse brain scRNA-seq data analysis’). Cell types with <50 cells were excluded from the analysis. Differential expression between conditions was assessed using the following two approaches: MAST^106^ on the cell level and edgeR’s likelihood ratio test^102,107^ on the sample level. For edgeR, a pseudobulk expression matrix was generated by aggregating single-cell counts per sample and cell type, and the analysis was performed with no intercept term and no additional covariates. Pseudobulks with ≤15 cells were excluded from the analysis. The expression of key marker genes and the full analysis pipeline are available at github.com/simonmfr/brain-vasc-scRNAseq-Foxf2-KO/tree/manuscript.

### Bulk RNA sequencing (bulk RNA-seq) of human iECs

#### RNA extraction, library preparation and sequencing

Cells were pelleted in culture and stored at −80 °C. RNA was extracted using Trizol (Qiagen, 79306) and the RNeasy mini kit (Qiagen, 74106) following the manufacturer’s instructions. A total of 400 ng of RNA was used for library construction as previously reported by BGI^108^.

#### Data analysis

We used SOAPnuke (29220494) to preprocess FASTQ files, including adaptor and low-quality trimming. Reads were further aligned to GRCh38 using bwa-mem2 (ref. ^109^). Normalization and differential gene expression analysis were performed using edgeR^110^.

### ChIP–seq

ChIP–seq was performed as previously described^111^. Briefly, two million cross-linked cells (1% formaldehyde, 10-min room temperature) were lysed in 100 μl buffer-B-0.3 (50 mM Tris–HCl, pH 8.0, 10 mM EDTA, 0.3% SDS, 1× protease inhibitors; Roche) and sonicated in a microtube (Covaris, 520045) using a Covaris S220 device until most of the DNA fragments were 200–500-bp long (settings—temperature 4 °C, duty cycle 2%, peak incident power 105 W, cycles per burst 200). After shearing, lysates were centrifuged (10 min, 4 °C, 12,000*g*) and supernatant diluted with 1 volume of dilution buffer (1 mM EGTA, 300 mM NaCl, 2% Triton X-100, 0.2% sodium deoxycholate, 1× protease inhibitors; Roche). Sonicated chromatin was then incubated for 4 h at 4 °C on a rotating wheel with 3 μg of anti-Flag antibody (Sigma-Aldrich, F1804) conjugated to 30 µl of Protein-G Dynabeads (Thermo Fisher Scientific). Beads were washed five times with buffer A (10 mM Tris–HCl, pH 7.5, 1 mM EDTA, 0.5 mM EGTA, 1% Triton X-100, 0.1% SDS, 0.1% sodium deoxycholate, 140 mM NaCl, 1× protease inhibitors) and once with buffer C (10 mM Tris–HCl, pH 8.0, 10 mM EDTA). Beads were then incubated with 70 μl elution buffer (0.5% SDS, 300 mM NaCl, 5 mM EDTA, 10 mM Tris–HCl, pH 8.0) containing 2 μl of proteinase K (20 mg ml^−1^) for 1 h at 55 °C and 8 h at 65 °C to revert formaldehyde crosslinking, and supernatant was transferred to a new tube. Another 30 μl of elution buffer was added to the beads for 1 min and eluates were combined and incubated with another 1 μl of proteinase K for 1 h at 55 °C. Finally, DNA was purified with solid phase reversible immobilization (SPRI) AMPure XP beads (Beckman Coulter) (sample-to-beads ratio 1:2). Purified DNA was used as the input for library preparation with Thruplex DNA-seq kit (Takara, R400674) and processed according to the manufacturer’s instructions. Libraries were quality controlled by Qubit and Agilent TapeStation analysis. Paired-end sequencing (60 bp) was performed on an Illumina NextSeq 2000 instrument.

The NGS pipeline (https://github.com/GunnarSchotta/NGS.analysis) with default settings was used for primary analysis and quality controls. Briefly, after adaptor trimming with Trimmomatic^112^ reads were mapped with Bowtie2^113^ to the hg38 human genome (parameters: --very-sensitive -X 2000). Following alignment read deduplication was carried out using Picard MarkDuplicates (Picard Toolkit, 2019; Broad Institute, GitHub Repository: https://broadinstitute.github.io/picard/; parameters: --VALIDATION_STRINGENCY LENIENT). Signal tracks for visualization with IGV genome browser (10.1038/nbt.1754) were generated with deepTools bamCoverage tool^114^ (parameters: –bam --binSize 10 --normalizeUsing RPKM). Homer^115^ findPeaks tool was used for peak calling (parameters: -style factor). Peaks common among replicates were identified using the Homer mergePeaks tool, and their genomic annotation was based on Homer annotatePeaks. Biological annotation of peaks was performed with the GREAT tool with default parameters^116^. Transcription factor motif analysis was performed using the MEME-ChIP suite^117^. H3K27ac ChIP–seq data were downloaded from ENCODE (https://www.encodeproject.org/experiments/ENCSR111QCU/).

#### Data analysis

We quantified binding regions per gene from our ChIP–seq dataset and normalized the counts to account for known biases. After lifting over binding region coordinates from hg38 to hg19 using the UCSC LiftOver tool^118^, gene annotations for hg19 were obtained from the GENCODE (v19) release, and a mappability track for 75-mer sequences was downloaded from the UCSC database. For each gene, its genomic coordinates and length were determined from the annotation. In addition to gene length, we computed the following two additional covariates: GC content and an average mappability score. The GC content was calculated by extracting the nucleotide sequence for each gene from the hg19 reference and determining the proportion of guanine and cytosine nucleotides. To calculate the average mappability score, we identified all intervals from the UCSC mappability track that overlapped the gene’s region. For each overlapping interval, we determined the length of the segment that intersected with the gene and multiplied that length by the corresponding mappability score. The sum of these weighted scores was then divided by the total length of all overlapping segments, yielding a length-weighted average mappability for each gene. These three factors—gene length, GC content and average mappability—were then incorporated into a Poisson model to estimate the expected number of binding regions per gene. We note that while our model captures major sources of bias, such as gene length, nucleotide composition and sequence alignability, additional factors (for example, chromatin accessibility, replication timing or other sequence features) may also influence the expected binding count. The observed binding counts were normalized by dividing by the model-derived expectations, and one-tailed *P* values (with Benjamini–Hochberg adjustment) were computed to assess enrichment. In a secondary model, we defined gene regulatory domains using a basal as well as an extension approach, in which the basal region was set as 5-kb upstream and 1-kb downstream of the transcription start site (strand-adjusted) and then extended outward to the midpoint between adjacent basal regions (bounded by chromosome ends)^116^. Binding events and mappability were recalculated over these extended domains, with the extended length used as an offset in a Poisson regression model.

### Assessment of vascular reactivity

Vascular reactivity was assessed as previously described^119^ in Foxf2^iECKO^ and Ctrl mice receiving vehicle or AKB-9778 treatment. Briefly, a 4-mm diameter chronic cranial window was implanted over the left somatosensory cortex. Three weeks after window implantation, mice were sedated with medetomidine (0.05 mg kg^−1^, subcutaneous) and isoflurane 0.5–0.75% (in air with 30% oxygen), fixed in a stereotactic frame and placed under an LSCI (Perimed) for the assessment of local CBF. Functional hyperemia was induced by manually stimulating the right whiskers five times for 40 s with a brush at a frequency of 1–2 Hz. Blood flow data were analyzed in an unbiased, investigator-independent manner using a custom-made MATLAB script (MATLAB, R2016b; The MathWorks).

One day after LSCI, the mice were re-anesthetized, and fluorescein isothiocyanate (FITC) dextran (2,000,000 kDa; Sigma-Aldrich) was administered intravenously to label the vessel lumen. Mice were placed under a two-photon microscope (7MP, Carl Zeiss AG) and pial and parenchymal vessels within the barrel cortex were visualized at a depth of 50–200 μm with a ×20 objective (EC Plan-NeoFluar, ×20, 1.0 NA; Carl Zeiss AG) and an 800 nm Li:Ti laser (Chameleon, Coherent). Whisker stimulation was performed with the same parameters as described above. Vessel diameters were determined in an unbiased, investigator-independent manner using a custom-made MATLAB routine (MATLAB, R2020a; The MathWorks).

### Experimental stroke model (MCAO)

For experimental stroke induction in Foxf2^iECKO^ and Ctrl mice, we performed transient MCAO as previously described in detail^120,121^. In brief, animals were anaesthetized with 2% isoflurane delivered in a mixture of 30% O_2_ and 70% N_2_O. The temporal bone was exposed by making an incision between the ear and the eye. In supine position, the mice were implanted with a laser Doppler probe that attached to the skull beyond the MCA territory. By performing a middle incision, the left common carotid artery and external carotid artery were exposed and further isolated and ligated. A 2-mm silicon-coated filament (Doccol) was inserted into the internal carotid artery, advanced gently to the MCA until resistance was felt, and occlusion was confirmed by a corresponding decrease in blood flow as shown in the laser Doppler flow signal by >80%. After 60 min of occlusion, the animals were re-anesthetized, and the filament was removed. Once the mice were awake, they were kept in their home cage with ad libitum access to water and food. In all mice, a feedback-controlled heating pad maintained the body temperature of 37 °C during surgery. Animals that (1) showed no sufficient MCAO (a decrease in blood flow to >20% of the baseline value), (2) died during surgery, (3) showed no ischemia on brain MRI scans or (4) had no or minimal focal neurological deficit (score 0 or 1) were excluded from the experiments.

### Neuroscore

The Neuroscore was performed 1 and 3 days after experimental stroke induction (1 and 3 dps). This test was used to evaluate the general status and focal neurologic dysfunction after transient ischemic attack and was performed as described before^122^. The score ranges from 0 (no deficits) to 44 (representing the poorest performance in all items) and is calculated as the sum of the general and focal deficits. The Neuroscore results were expressed as a composite neurological score, which included the following general deficits (scores): fur (0–2), ears (0–2), eyes (0–4), posture (0–4), spontaneous activity (0–4); and the following focal deficits: body asymmetry (0–4), gait (0–4), climbing on a surface inclined at 45° (0–4), circling behavior (0–4), front-limb symmetry (0–4), compulsory circling with front limbs on the bench (0–4) and whisker response to light touch (0–4).

### MRI

MRI was performed in a 3T nanoScan PET/MR 3T scanner equipped with a surface coil optimized for the mouse head (Mediso), 1 and 3 days after stroke surgery. For scanning, mice were anesthetized with 1.2% isoflurane in 30% O_2_ and 70% N_2_O applied using a face mask. Respiratory rate and body temperature (37±0.5°C) were continuously monitored through an abdominal pressure-sensitive pad and anesthesia was adjusted to keep them in a physiological range. Imaging data were obtained using a coronal T2 fast spin-echo (T2FSE) weighted sequence (acquisition time, 0:07:38; slices, 22; number of excitations (NEX), 4; repetition time (TR), 10,911; echo time (TE), 66.3; averages, 4). Here 3D-stack MRI images were processed in ImageJ software (v1.52p). Infarct volumes were determined by eight consecutive coronal slices and expressed as a percentage (%) of brain volume.

### Pharmacological treatments

#### AKB-9778 treatment in vivo

Mice were intraperitoneally injected four times with 30 mg kg^−1^ of AKB-9778 (30 mg kg^−1^) or PBS (Vehicle or Veh) as a control every 12 h as previously reported^63^.

#### AKB-9778, angiopoietin-1 (ANGPT1) and Bradykinin treatment of human iECs

Cells were seeded onto Collagen IV-coated culture plates and treatment was started when cells reached confluency. Cells were treated with 1 µM AKB-9785 diluted in DMSO and Mygliol (AKB; MedChem, HY-109041) for 24 h, 2 µg ml^−1^ ANG1 diluted in H_2_O (MedChem, HY-P70061) for 96 h or 20 µM and 200-µM Bradykinin diluted in acetic acid (Enzo Life Sciences, ALX-152-006-M005) for 30 min. Corresponding Veh solution (dilution media without pharmacological specimen) was always applied to a control group. Cells were either fixed for imaging or lysed on ice for protein and RNA analysis.

### Statistical analysis

Sample sizes were determined by results obtained in previous proteomic and immunohistochemical studies on brain vessels and BECs discussed in refs. ^53,89,90^. Animal-based experiments included three to eight animals per genotype. Animals for pharmacological and vehicle treatment were randomly selected after genotyping. Blinding was applied to in vivo experiments (surgery and recording), tissue processing (BEC and vessel preparation), microscopy and image analysis. In vitro experiments included three to four samples per group. Proteomic, transcriptomic and morphological datasets showed normal distribution (tested with GraphPad Prism (v10.4)). Statistical significance was analyzed using GraphPad Prism, except for proteomics analysis, where Perseus (v1.6.2.3) and Excel (v16.9) were used. Significance was analyzed by two-tailed unpaired *t* test unless stated otherwise (*P* values and group sizes are indicated in each experiment). Perseus was used to apply a permutation-based FDR estimation for multiple hypotheses. Multiple comparisons were corrected as recommended by GraphPad Prism using Tukey’s method. For qualitative analysis, experiments were repeated at least thrice. All data values of descriptive statistics are given as mean ± s.d. with *P < 0.05; **P < 0.01; ***P < 0.001 unless stated otherwise. Statistical details are also specified in the figure legends.

### Reporting summary

Further information on research design is available in the Nature Portfolio Reporting Summary linked to this article.

## Online content

Any methods, additional references, Nature Portfolio reporting summaries, source data, extended data, supplementary information, acknowledgements, peer review information; details of author contributions and competing interests; and statements of data and code availability are available at 10.1038/s41593-025-02136-5.

## Supplementary information

Supplementary Information Supplementary Tables 9 and 10.

Reporting Summary

Supplementary Table 1 All quantified mRNAs from scRNA-seq of BECs from Foxf2^iECKO^ and Ctrl mice. Green color indicates significant changes in mRNA abundance in Foxf2^iECKO^ versus Ctrl mice (*n* = 6 mice per group pooled into *n* = 3 samples per condition, comparison by MAST and edgeR, *P* < 0.05).

Supplementary Table 2 Vessel metrics and EB leakage analysis of whole brain vasculature of Foxf2^iECKO^ mice using VesSAP. Red color in column N indicates significantly altered vessel metrics by anatomical regions in Foxf2^iECKO^ versus Ctrl mice (*n* = 4 mice per group, comparison by two-tailed unpaired *t* test, *P* < 0.05).

Supplementary Table 3 All quantified mRNAs from bulk RNA-seq of iECs from FOXF2^KO^ and WT cells. Green and yellow colors indicate significant changes in mRNA abundance in FOXF2^KO^ versus WT iECs (*n* = 4 iEC samples per group, the number of iEC samples reflects technical replicates; differential gene expression was tested using a negative binomial generalized linear model, *P* < 0.05).

Supplementary Table 4 Target gene candidates of FOXF2 based on the ChIP–seq of human iECs. **a**, Columns indicate the gene name, the position of associated FOXF2 peaks relative to the transcription start site and the number of peaks that associate with each gene. Top FOXF2-bound genes (≥10 peaks) are highlighted in yellow. **b**, Columns indicate the gene and length, mappability, binding density, number of binding sites, normalized ratio, *P* value and adjusted *P* value using a Poisson model. Genes that showed a significant enrichment compared to the values expected from a Poisson model are highlighted in yellow. **c**, Columns indicate the gene and length, mappability, number of binding sites, normalized ratio, *P* value and adjusted *P* value using a Poisson model. Genes that showed a significant enrichment compared to the values expected from the Poisson model are highlighted in yellow.

Supplementary Table 5 Autopsy sample characteristics from patients with sporadic cerebral SVD and control participants.

Supplementary Table 6 All quantified proteins of BECs from Foxf2^iECKO^ and Ctrl mice. Red color in column E indicates significant changes in protein abundance in Foxf2^iECKO^ versus Ctrl mice (*n* = 6 mice per group, comparison by two-tailed unpaired *t* test, *P* < 0.05).

Supplementary Table 7 All quantified proteins of brain vessels from vehicle and AKB-9778-treated Foxf2^iECKO^ and Ctrl mice. **a**, Red color in column G indicates significant changes in protein abundance in Foxf2^iECKO^-Veh versus Ctrl-Veh or Foxf2^iECKO^-AKB versus Foxf2^iECKO^-Veh mice. **b**, Red color in column E indicates significant changes in protein abundance in Ctrl-Veh versus Ctrl-AKB mice (*n* = 4 mice per group, comparison by two-tailed unpaired *t* test, *P* < 0.05 (**a**,**b**)).

Supplementary Table 8 All quantified proteins of human iECs from vehicle and AKB-9778-treated FOXF2^KO^ and WT cells. Red color in columns E and G indicates significant changes in protein abundance in FOXF2^KO^-Veh versus WT-Veh or FOXF2^KO^-AKB versus FOXF2^KO^-Veh iECs (*n* = 4 iEC samples per group, the number of iEC samples reflects technical replicates, comparison by two-tailed unpaired *t* test, *P* < 0.05).

## Source data

Source Data Fig. 1 FOXF2 acts as a transcriptional activator of cell-adhesion-related and angiogenesis-related genes, including TIE2. **c**, mRNA abundance of the most affected angiogenesis-related and cell-adhesion-related receptors in BECs of FOXF2^iECKO^ versus Ctrl mice. **d**, Relative mRNA abundance of Tie2 and Nos3 transcripts in full brain tissue of FOXF2^iECKO^ versus Ctrl mice. **e**, Relative mRNA abundance of Foxf2, Foxc1, Foxq1 and Foxo1 transcripts in full brain tissue of FOXF2^iECKO^ versus Ctrl mice. Comparison by two-tailed unpaired *t* test, *n* = 4–6 mice per group. **i**, mRNA abundance of the most affected angiogenesis-related and cell-adhesion-related receptors in FOXF2^KO^ versus WT iECs. **j**, Relative mRNA abundance of TIE2 and NOS3 transcripts in FOXF2^KO^ versus WT iECs. **k**, Relative protein abundance of TIE2 and NOS3 in FOXF2KO versus WT iECs (normalized to PECAM1). **l**, Relative mRNA abundance of VEGFR2 angiogenesis marker in FOXF2^KO^ versus WT iECs. **n**, Relative mRNA abundance of FOXF2 in FOXF2 overexpressing versus WT iECs. Data are presented as mean ± s.d., comparison by two-tailed unpaired *t* test (**d**,**e**,**j**–**l**,**n**). *n* = 4 mice per group (**d**). Foxf2, Foxq1, Foxo1, *n* = 6 mice per group; Foxc1, *n* = 5 Ctrl and *n* = 4 iECKO mice per group (**e**). *n* = 5 WT and *n* = 6 KO iEC samples per group (**j**). *n* = 4 iEC samples per group (**k**,**l**). *n* = 6 WT and *n* = 11 FOXF2-overexpressing iEC samples per group (**n**). The number of iEC samples reflects technical replicates (**j**–**n**).

Source Data Fig. 2 Endothelial Foxf2 deficiency causes BBB leakage and attenuates Tie2 signaling. **c**, Quantification of the extravasation of tracers (EB—65 kDa, TMR-dextran—40 kDa, CB-dextran—10 kDa, A555-cadaverine—1 kDa). **d**, Quantification of vessel density and fibrinogen extravasation in histopathological sections from SVD patients and Ctrls (*n* = 6 patients per group). **i**, Subcellular localization of significantly dysregulated proteins. **l**, Abundance of significantly downregulated proteins according to top-enriched Tie2-regulated biological processes, and of significantly upregulated proteins related to reactive oxygen species metabolic process and cellular response to oxidative stress. **m**, Abundance of significantly altered selected proteins according to the top-enriched biological processes. Data are presented as mean ± s.d., comparison by two-tailed unpaired *t* test (**c**, **d** and **m**). *n* = 3 Ctrl-, EB and Dxt40: *n* = 3 iECKO-, Dxt10 and Cad1: *n* = 4 iECKO mice per group (**c**). *n* = 6 mice per group (**m**).

Source Data Fig. 3 Endothelial Foxf2 facilitates functional hyperemia and limits infarct size in adult mice via Tie2 signaling. **c**, Abundance of Tie2–Nos3 signaling-related proteins that were rescued by treatment with AKB-9778. **d**, Quantification of Nos3 labeling in isolated brain microvessels. **e**, Quantification of mean CBF changes within Barrel cortex obtained by LSCI as well as vessel diameter changes of penetrating arterioles and capillaries obtained by two-photon microscopy (2P) following whisker stimulation. **f**, Quantification of infarct size and Alb leakage in mice treated with either vehicle or AKB-9778 before fMCAO. **g**,**h**, Quantification of Tjp1 labeling in the contralateral cortex 24 h after stroke (**g**) and in peri-infarct regions of the ipsilateral cortex (**h**). **i**,**j**, Quantification of glial endfeet (Aqp4) (**i**) and neurons (NeuN) (**j**) in the cortical regions of the infarct area, peri-infarct regions of the ipsilateral cortex, and corresponding regions of the contralateral cortex 24 h after stroke. Comparison by two-tailed unpaired *t* test, *P* < 0.05 (**c**–**j**). *n* = 4 mice per group (**c**). *n* = 24 images per group (**d**). CBF, *n* = 5 Ctrl-Veh^−^, *n* = 6 iECKO-Veh^−^, and *n* = 6 iECKO-AKB mice per group; vessel diameter, *n* = 4 mice per group (**e**). *n* = 6 mice per group (**f**). *n* = 12 images per group (**g**, **h**, top; **i** and **j**).

Source Data Fig. 4 AKB-9778 restores TIE2 signaling and NO production in human endothelial cells lacking FOXF2. **c**, Abundance of TIE2-signaling-related significantly altered (FOXF2KO-Veh versus WT-Veh) and rescued proteins upon AKB-9778 treatment (FOXF2KO-AKB versus FOXF2KO-Veh). **d**, Quantification of NOS3 in vehicle-treated WT, and vehicle or AKB-treated FOXF2KO iECs. **e**, Western blot quantification of pAkt/Akt in naive, vehicle or AKB-treated iECs. **f**,**h**, Top, quantification of pTIE2 (**f**) and pFOXO1 (**h**, top) in vehicle or AKB-treated iECs. **g**, Quantification of NO production in vehicle or Bradykinin-treated iECs by DAF-2T fluorescence. **h**, Bottom, quantification of NO production in vehicle or AKB-treated iECs by DAF fluorescence. **i**,**j**, Top, quantification of pNOS3 (**i**) and pFOXO1 (**j**) in vehicle or Ang1-treated iECs. **j**, Bottom, quantification of NO production in vehicle or Ang1-treated iECs by DAF fluorescence. Comparison by two-tailed unpaired *t* test (**c**–**f**, **h**, **i**, **j**). *n* = 4 iEC samples per group (**c** and **e**). *n* = 10 iEC images per group (**d** and **h**, top). *n* = 13 WT-Veh, *n* = 9 KO-Veh and *n* = 13 KO-AKB iEC images per group (**f**). *n* = 14 iEC images per group (**g**, **h**, bottom; **i** and **j**, bottom). *n* = 15 iEC images per group; the exact *P* values are presented in **j** (top). The number of iEC samples reflects technical replicates (**c**–**j**).

## Data Availability

The mass-spectrometry proteomics data have been deposited to the ProteomeXchange Consortium using the PRIDE^123^ partner repository with the dataset identifiers PXD051838, PXD051839 and PXD051855. Transcriptomics and ChIP–seq datasets are available at GEO with the accessions GSE265959 and GSE265820, respectively. All data supporting the findings described in this manuscript are available in the article and in Supplementary Information and from the corresponding author upon request. Source data are provided with this paper.

## Code availability

No custom software codes were used to analyze the data.
