# Supplementary material for: The stroke risk gene Foxf2 maintains brain endothelial cell function via Tie2 signaling
Source: Nat Neurosci. 2025 Dec 15;29(2):325–36. doi: 10.1038/s41593-025-02136-5 (PMC12880920; doi:10.1038/s41593-025-02136-5)
Supplement: Supplementary file 1 — Supplementary Tables 9 and 10. [file 41593_2025_2136_MOESM1_ESM.pdf]

nature neuroscience

Supplementary information

https://doi.org/10.1038/s41593-025-02136-5

The stroke risk gene *Foxf2* maintains brain endothelial cell function via Tie2 signaling

In the format provided by the authors and unedited

Antibody	Species	Source	Catalog Number	Dilution	
				ICC / IHC	WB
**Primary antibodies**					
Akt	Rabbit	Cell Signaling	9272		1:250
Alb	Mouse	Sigma	A6684	1:200	
Cdh5	Goat	R&D system	AF938	1:150	
Col4	Goat	Southern Biotech	1340-01	1:400	
EMC7	Mouse	Santa Cruz	SC-514440		1:250
Fg	Rabbit	Dako	AF3628	1:100	
NeuN	Mouse	Millipore	A0080	1:200	
Nos3	Rabbit	Abcam	Ab5589	1:200	
pAkt	Rabbit	Cell Signaling	4060	1:100	1:250
Pecam1	Rabbit	Cell Signaling	77699		1:250
Pecam1	Goat	R&D system	AF3628	1:100	1:250
pFoxo1	Rabbit	Cell Signaling	9461	1:100	
pNos3	Rabbit	Abcam	ab215717	1:100	
pTie2	Rabbit	R&D system	AF2720	1:100	
Tie2	Mouse	Cell Signaling	4224S		1:250
**Secondary antibodies**					
Alexa-488	Mouse	Jackson Laboratories	715-546-150	1:500	
Alexa-488	Rabbit	Jackson Laboratories	711-545-152	1:500	
Alexa-488	Goat	Jackson Laboratories	705-546-147	1:500	
Alexa-647	Mouse	Jackson Laboratories	715-606-150	1:500	
Alexa-647	Rabbit	Jackson Laboratories	711-606-152	1:500	
Alexa-647	Goat	Jackson Laboratories	705-606-147	1:500	
Cy3	Mouse	Jackson Laboratories	715-165-150	1:500	
Cy3	Rabbit	Jackson Laboratories	711-165-152	1:500	
Cy3	Goat	Jackson Laboratories	705-165-147	1:500	
**HRP conjugated antibodies**					
HRP	Mouse	Dako	P0447		1:10000
HRP	Rabbit	Dako	F026102-2		1:10000

Suppl. Table 9. Primary and secondary antibodies for the experiments in the present study.
ICC, immunocytochemistry; IHC, immunohistochemistry; WB, Western blot.

Gene	Forward primer (5´-3´)	Reverse primer (5´-3´)
EMC7	AAAGGAGGTAGTCAGGCCGT	GTTGCTTCACACGGTTTTCCA
Foxc1	GCTTCAGCGTGGACAACATC	GGAACCGAGCTCAGCGG
Foxc2	CATGTTCGAGAATGGCAGCTT	TTGGCCGTGGTCGAGGG
Foxf1	AACCCAGCCGCCAACC	CCGAGGGATGCCTTGCAG
Foxf2	GCGAGGATCTCTCAGTCGG	AGCGGAAGGGTGGAAAGAAG
FOXF2	GCTCAAGCCCATGTACCACC	CCTGGAAGTCGAAGCCCTG
Foxo1	AAGAGTTAGTGAGCAGGCTACAT	GGACTGCTCCTCAGTTCCTG
Foxq1	AGACCGATAGCATAAGCGCC	CTTTGAGCGGAAGACAAGCG
Gapdh	GCCTCAAGATCATCAGC	ACCACTGACACGTTGGC
Nos3	TCTACCGGGACGAGGTACTG	TCTTGCACGTAGGTCTTGGG
NOS3	GCCGGAACAGCACAAGAGTTA	CCCTGCACTGTCTGTGTTACT
Tie2	ACCGTGGACAGGGGAGATAA	TGAGTGGATGAAGGAGCCATT
TIE2	ACTGTGCTGTTGGCCTTTCT	GCTGGTTCTTCCCTCACGTT
VEGFR2	GGAACCTCACTATCCGCAGAGT	CCAAGTTCGTCTTTTCCTGGGC

Supplementary Table 10. Primer sequences for the experiments in the present study.
