# Supplementary material for: The stroke risk gene Foxf2 maintains brain endothelial cell function via Tie2 signaling
Source: Nat Neurosci. 2025 Dec 15;29(2):325–36. doi: 10.1038/s41593-025-02136-5 (PMC12880920; doi:10.1038/s41593-025-02136-5)
Supplement: Supplementary file 2 — Reporting Summary [file 41593_2025_2136_MOESM2_ESM.pdf]

nature portfolio

Corresponding author(s): Martin Dichgans and Dominik Paquet

Last updated by author(s): 13.09.2025

Reporting Summary

Nature Portfolio wishes to improve the reproducibility of the work that we publish. This form provides structure for consistency and transparency in reporting. For further information on Nature Portfolio policies, see our Editorial Policies and the Editorial Policy Checklist.

Statistics

For all statistical analyses, confirm that the following items are present in the figure legend, table legend, main text, or Methods section.

n/a	Confirmed	
☐	☒	The exact sample size (*n*) for each experimental group/condition, given as a discrete number and unit of measurement
☐	☒	A statement on whether measurements were taken from distinct samples or whether the same sample was measured repeatedly
☐	☒	The statistical test(s) used AND whether they are one- or two-sided<br>*Only common tests should be described solely by name; describe more complex techniques in the Methods section.*
☒	☐	A description of all covariates tested
☐	☒	A description of any assumptions or corrections, such as tests of normality and adjustment for multiple comparisons
☐	☒	A full description of the statistical parameters including central tendency (e.g. means) or other basic estimates (e.g. regression coefficient) AND variation (e.g. standard deviation) or associated estimates of uncertainty (e.g. confidence intervals)
☐	☒	For null hypothesis testing, the test statistic (e.g. *F*, *t*, *r*) with confidence intervals, effect sizes, degrees of freedom and *P* value noted<br>*Give P values as exact values whenever suitable.*
☒	☐	For Bayesian analysis, information on the choice of priors and Markov chain Monte Carlo settings
☒	☐	For hierarchical and complex designs, identification of the appropriate level for tests and full reporting of outcomes
☒	☐	Estimates of effect sizes (e.g. Cohen's *d*, Pearson's *r*), indicating how they were calculated

Our web collection on statistics for biologists contains articles on many of the points above.

Software and code

Policy information about availability of computer code

Data collection	Zeiss Confocal microscope (LSM880 and LSM980) using 10x, 40x and 63x objectives; laser speckle contrast imager (LSCI, Perimed, Järfälla, Sweden); two-photon microscope (7MP, Carl Zeiss AG, Germany); LaVision UltraII microscope coupled to a white light laser module (NKT SuperK Extreme EXW-12); Fusion FX7 (Vilber Lourmat); Crossbeam Gemini 340 SEM (Zeiss); Illumina HiSeq4000; 3T nanoScan PET/MR 3T scanner equipped with a surface coil optimized for the mouse head (Mediso, Hungary); nanoElute nanoHPLC which was coupled to a TimsTOF pro mass spectrometer with a CaptiveSpray ion source (Bruker, Germany); BD FACSAriaIII.
Data analysis	Maxquant (version 1.6.17.), DIA-NN (Version 1.8), and DAVID (v2023q4) analysis of proteomic data and statistics; ImageJ (version 1.52p) for image and gel analysis; Fiji TrakEM2 (Ref 91) for serial EM section analysis; FACSDiva version 8.0.1 for flow cytometry; VesSAP pipeline (Ref: 45); Python (version 3.9) and R (version 4.0.0) for single-cell data analysis; NGS pipeline (https://github.com/GunnarSchotta/NGS.analysis) for ChIP-seq analysis; MATLAB (R2016b and R2020a) for blood flow data analysis, GraphPad (8.3.1), Excel (2016), and Adobe Illustrator (2017) for statistics and figure representation.

For manuscripts utilizing custom algorithms or software that are central to the research but not yet described in published literature, software must be made available to editors and reviewers. We strongly encourage code deposition in a community repository (e.g. GitHub). See the Nature Portfolio guidelines for submitting code & software for further information.

Data

Policy information about availability of data

All manuscripts must include a data availability statement. This statement should provide the following information, where applicable:

- Accession codes, unique identifiers, or web links for publicly available datasets
- A description of any restrictions on data availability
- For clinical datasets or third party data, please ensure that the statement adheres to our policy

All proteomic, transcriptomic, and ChIP-seq data are available for the reviewers through public repositories with the following accession codes:

Proteomics:

Project Name: Mass spectrometry analysis of brain endothelial cells (BECs) from Foxf2iECKO and Ctrl mice
Project accession: https://www.ebi.ac.uk/pride/archive/projects/PXD051838

Project Name: Mass-spectrometry analysis of isolated brain vessels from vehicle treated Foxf2iECKO, vehicle-treated Ctrl, and AKB-9778 treated Foxf2iECKO mice
Project accession: https://www.ebi.ac.uk/pride/archive/projects/PXD051839

Project Name: Mass spectrometry analysis of isolated brain vessels from vehicle treated Foxf2iECKO, vehicle-treated Ctrl, and AKB-9778 treated Foxf2iECKO mice
Project accession: https://www.ebi.ac.uk/pride/archive/projects/PXD051855

Transcriptomics

Project Name: Single cell RNA sequencing (scRNAseq) of Foxf2iECKO vs Ctrl mouse brain endothelial cell (BEC):
Project accession: https://www.ncbi.nlm.nih.gov/geo/query/acc.cgi?acc=GSE265959

ChIP-sequencing

Project Name: Chromatin Immunoprecipitation sequencing (ChIP-seq) of FOXF2 overexpressing human endothelial cells
Project accession: https://www.ncbi.nlm.nih.gov/geo/query/acc.cgi?acc=GSE265820

Research involving human participants, their data, or biological material

Policy information about studies with human participants or human data. See also policy information about sex, gender (identity/presentation), and sexual orientation and race, ethnicity and racism.

Reporting on sex and gender	Sex and gender analysis was not considered in this study due to low n number. Sex was self reported.
Reporting on race, ethnicity, or other socially relevant groupings	N/A
Population characteristics	Cerebral small vessel disease (SVD) and control subjects were age- and sex-matched (see Suppl. Table 5 for additional information).
Recruitment	Samples were obtained from the Netherlands Brain Bank (Netherlands Institute for Neuroscience, Amsterdam; www.brainbank.nl)
Ethics oversight	All material has been collected from donors for whom a written informed consent for a brain autopsy and the use of the material and clinical information for research purpose had been obtained.

Note that full information on the approval of the study protocol must also be provided in the manuscript.

Field-specific reporting

Please select the one below that is the best fit for your research. If you are not sure, read the appropriate sections before making your selection.

☒ Life sciences ☐ Behavioural & social sciences ☐ Ecological, evolutionary & environmental sciences

For a reference copy of the document with all sections, see nature.com/documents/nr-reporting-summary-flat.pdf

Life sciences study design

All studies must disclose on these points even when the disclosure is negative.

Sample size	Sample sizes were determined based on results obtained in previous proteomic and immunohistochemical studies on brain vessels and brain endothelial cells (BECs) published by the authors (Ref 49 and 80).

Data exclusions	Animals with < 70% Foxf2 deletion efficiency were excluded from further analysis (n=3). In the MCAo experiments animals that i) showed no sufficient MCA occlusion (a decrease in blood flow to > 20% of the baseline value); ii) died during surgery, or iii) that showed no ischemia on brain MRI scans were excluded from the experiments. Exclusion of cells in scRNA-seq analyses were conducted based on QC metrics and are specified in the methods section.
Replication	Animal-based experiments included 3-8 animals per genotype, In vitro experiments included 3-4 samples per group.
Randomization	Animals for pharmacological and vehicle treatment were randomly selected after genotyping.
Blinding	Blinding was applied to in vivo experiments (surgery and recording), tissue processing (BEC and vessel preparation), microscopy, and image analysis.

Reporting for specific materials, systems and methods

We require information from authors about some types of materials, experimental systems and methods used in many studies. Here, indicate whether each material, system or method listed is relevant to your study. If you are not sure if a list item applies to your research, read the appropriate section before selecting a response.

Materials & experimental systems

n/a	Involved in the study	
☐	☒	Antibodies
☐	☒	Eukaryotic cell lines
☒	☐	Palaeontology and archaeology
☐	☒	Animals and other organisms
☒	☐	Clinical data
☒	☐	Dual use research of concern
☒	☐	Plants

Methods

n/a	Involved in the study	
☐	☒	ChIP-seq
☐	☒	Flow cytometry
☐	☒	MRI-based neuroimaging

Antibodies

Antibodies used	Akt, Rabbit, Cell Signaling, 9272, 1:250 dilution<br>Alb, Mouse, Sigma, A6684, 1:200 dilution<br>Cdh5, Goat, R&D system, AF938, 1:150 dilution<br>Col4, Goat, Southern Biotech, 1340-01, 1:400 dilution<br>EMC7, Mouse, Santa Cruz, SC-514440, 1:250 dilution<br>Fg, Rabbit, Dako, AF3628, 1:100 dilution<br>NeuN, Mouse, Millipore, A0080, 1:200 dilution<br>Nos3, Rabbit, Abcam, Ab5589, 1:200 dilution<br>pNos3, Rabbit, Abcam, Ab215717, 1:100 dilution<br>pAkt, Rabbit, Cell Signaling, 4060, 1:100 / 1:250 dilution<br>Pecam1, Rabbit, Cell Signaling, 77699, 1:250 dilution<br>Pecam1, Goat, R&D system, AF3628, 1:100 / 1:250 dilution<br>pFoxo1, Rabbit, Cell Signaling, 9461, 1:200 dilution<br>pTie2, Rabbit, R&D system, AF2720, 1:200 dilution<br>Tie2, Mouse, Cell Signaling, 4224S, 1:250 dilution<br>Tjp1, Mouse, Thermo Fischer, 33-9100, 1:100 dilution<br>A488, Mouse, Jackson Laboratories, 715-546-150, 1:500 dilution<br>A488, Rabbit, Jackson Laboratories, 711-545-152, 1:500 dilution<br>A488, Goat, Jackson Laboratories, 705-546-147, 1:500 dilution<br>A647, Mouse, Jackson Laboratories, 715-606-150, 1:500 dilution<br>A647, Rabbit, Jackson Laboratories, 711-606-152, 1:500 dilution<br>A647, Goat, Jackson Laboratories, 705-606-147, 1:500 dilution<br>Cy3, Mouse, Jackson Laboratories, 715-165-150, 1:500 dilution<br>Cy3, JRabbit, ackson Laboratories, 711-165-152, 1:500 dilution<br>Cy3, Goat, Jackson Laboratories, 705-165-147, 1:500 dilution<br>HRP, Mouse and Rabbit, Dako, P0447 and F026102-2, 1:10.000 dilution
Validation	All Abs are commercially available and were used according to the manufacturer´s instructions.

Eukaryotic cell lines

Policy information about [cell lines and Sex and Gender in Research]

Cell line source(s)	A18944: purchased from Thermofisher (Cat. No. A18945)
Authentication	Authenticated by manufacturer.

Mycoplasma contamination	The line was regularly tested and confirmed negative for mycoplasma contamination.
Commonly misidentified lines (See ICLAC register)	N/A

Animals and other research organisms

Policy information about studies involving animals; ARRIVE guidelines recommended for reporting animal research, and Sex and Gender in Research

Laboratory animals	Mus musculus, Foxf2fl/fl;Cdh5-Cre (Foxf2iECKO) and Foxf2fl/fl (Ctrl) lines with C57BL/6J background, 6 month old
Wild animals	The study did not involve wild animals.
Reporting on sex	Mixed-sex groups with the same ratio of male and female mice were used for all experiments.
Field-collected samples	The study did not involve samples collected from the field.
Ethics oversight	All mouse-based experiments were performed in accordance with the German Animal Welfare Law and in compliance with the Government of Upper Bavaria.

Note that full information on the approval of the study protocol must also be provided in the manuscript.

Plants

Seed stocks	*Report on the source of all seed stocks or other plant material used. If applicable, state the seed stock centre and catalogue number. If plant specimens were collected from the field, describe the collection location, date and sampling procedures.*
Novel plant genotypes	*Describe the methods by which all novel plant genotypes were produced. This includes those generated by transgenic approaches, gene editing, chemical/radiation-based mutagenesis and hybridization. For transgenic lines, describe the transformation method, the number of independent lines analyzed and the generation upon which experiments were performed. For gene-edited lines, describe the editor used, the endogenous sequence targeted for editing, the targeting guide RNA sequence (if applicable) and how the editor was applied.*
Authentication	*Describe any authentication procedures for each seed stock used or novel genotype generated. Describe any experiments used to assess the effect of a mutation and, where applicable, how potential secondary effects (e.g. second site T-DNA insertions, mosiacism, off-target gene editing) were examined.*

ChIP-seq

Data deposition

☒ Confirm that both raw and final processed data have been deposited in a public database such as GEO.

☒ Confirm that you have deposited or provided access to graph files (e.g. BED files) for the called peaks.

Data access links<br>*May remain private before publication.*	Reviewing of deposited data still in process. GEO accession numbers and link to deposited data will be available within five business days. The bed file of the peaks common between replicates is included in the supplementary files.
Files in database submission	For each ChIP sample the GEO entry contains the raw data (fastq format) and a file with coverage track (bigWig format) for visualization in a genome browser.<br><br>fastq files:<br>read1_GS1765.fastq.gz<br>read1_GS1766.fastq.gz<br>read2_GS1765.fastq.gz<br>read2_GS1766.fastq.gz<br><br>bigWig files:<br>E_Foxf2-Flag_ChIP_r1.dedup.unique.bw<br>E_Foxf2-Flag_ChIP_r2.dedup.unique.bw
Genome browser session<br>(e.g. UCSC)	The following coverage tracks (bigWig files) have been deposited in GEO and can be used for visualization with IGV genome browser (https://doi.org/10.1038/nbt.1754):<br><br>E_Foxf2-Flag_ChIP_r1.dedup.unique.bw<br>E_Foxf2-Flag_ChIP_r2.dedup.unique.bw

Methodology

Replicates	2

Sequencing depth	Paired-end sequencing (60bp) was performed on an Illumina Next-Seq 2000 instrument. Raw reads and mapped reads after filtering are given below:<br><br>sample_name Raw_reads Mapped_reads_filtered<br>E_Foxf2-Flag_ChIP_r1 27635640 23117814<br>E_Foxf2-Flag_ChIP_r2 24913328 20815231
Antibodies	Anti-Flag M2 Sigma F1804
Peak calling parameters	Homer findPeaks tool was used for peak calling (parameters: -style factor). Peaks common between replicates were identified using the homer mergePeaks tool. Only peaks common between replicated were retained for further analysis.
Data quality	ChIPseq sample quality was assessed using the following criteria:<br>Technical quality (sufficient sequencing depth and mapping rates)<br>Reproducibility (high person correlation between replicates)<br>Specificity (enrichment of the Foxf2 motif in called peaks)
Software	The NGS pipeline (https://github.com/GunnarSchotta/NGS.analysis) with default settings was used for primary analysis and quality controls. Coverage tracks were generated with deepTools (PMID: 27079975). Homer (PMID: 20513432) was used for peak calling. Biological annotation of peaks was performed with the GREAT tool with default parameters (PMID: 20436461). Transcription factor motif analysis was done with the MEME-ChIP suite (PMID: 21486936).

Flow Cytometry

Plots

Confirm that:

☐ The axis labels state the marker and fluorochrome used (e.g. CD4-FITC).

☐ The axis scales are clearly visible. Include numbers along axes only for bottom left plot of group (a 'group' is an analysis of identical markers).

☐ All plots are contour plots with outliers or pseudocolor plots.

☐ A numerical value for number of cells or percentage (with statistics) is provided.

Methodology

Sample preparation	BECs were isolated from whole mouse brain as previously described (Ref 49).
Instrument	BD FACSAriaIII
Software	FACSDiva version 8.0.1
Cell population abundance	Sorted population P4 -> 3% of parental
Gating strategy	All population > singlets > Alive (using ixable Viability Dye eFluor™ 780 (Invitrogen, Cat# 65-0865-14) > microglia negative (using CD11b (Invitrogen, Cat# 53-0112-82) + CD45 (Invitrogen, Cat# 53-1080 0451-82)).

☐ Tick this box to confirm that a figure exemplifying the gating strategy is provided in the Supplementary Information.

Magnetic resonance imaging

Experimental design

Design type	Fixed interval imaging after stroke.
Design specifications	Magnetic resonance imaging was performed in a 3T nanoScan PET/MR 3T scanner equipped with a surface coil optimized for the mouse head (Mediso, Hungary) 1 and 3 days after stroke surgery. For scanning, mice were anesthetized with 1.2% isoflurane in 30% O2 and 70% N2O applied via face mask. Respiratory rate and body temperature (37 +/- 0,5 °C) were continuously monitored via an abdominal pressure sensitive pad and anaesthesia adjusted to keep them in a physiological range. Imaging data were obtained using a coronal T2 fast spin-echo (T2FSE) weighted sequence (Acquisition time: 0:07:38, Slices: 22, NEX: 4, TR: 10911, TE: 66.3, averages: 4). 3D-stack MRI images were processed in ImageJ software (version 1.52p). Infarct volumes were determined on 8 consecutive coronal slices and expressed as percent (%) of brain volume.
Behavioral performance measures	Prior to MRI, mice were subjected to Neuroscore assessments to evaluate both general condition and focal neurological deficits.

Acquisition

Imaging type(s): Structural

Field strength: Small animal scanner (3T nanoScan® PET/MR, Mediso, with 25 mm internal diameter quadrature mouse head coil).

Sequence & imaging parameters: Coronal T2 fast spin-echo (T2FSE) weighted sequence (Acquisition time: 0:07:38, Slices: 22, NEX: 4, TR: 10911, TE: 66.3, averages: 4)

Area of acquisition: Mouse brain

Diffusion MRI: ☐ Used ☒ Not used

Preprocessing

Preprocessing software: Nucline nano Scan 3.04.014.0000; ImageJ software

Normalization: *If data were normalized/standardized, describe the approach(es): specify linear or non-linear and define image types used for transformation OR indicate that data were not normalized and explain rationale for lack of normalization.*

Normalization template: *Describe the template used for normalization/transformation, specifying subject space or group standardized space (e.g. original Talairach, MNI305, ICBM152) OR indicate that the data were not normalized.*

Noise and artifact removal: *Describe your procedure(s) for artifact and structured noise removal, specifying motion parameters, tissue signals and physiological signals (heart rate, respiration).*

Volume censoring: *Define your software and/or method and criteria for volume censoring, and state the extent of such censoring.*

Statistical modeling & inference

Model type and settings: *Specify type (mass univariate, multivariate, RSA, predictive, etc.) and describe essential details of the model at the first and second levels (e.g. fixed, random or mixed effects; drift or auto-correlation).*

Effect(s) tested: *Define precise effect in terms of the task or stimulus conditions instead of psychological concepts and indicate whether ANOVA or factorial designs were used.*

Specify type of analysis: ☒ Whole brain ☐ ROI-based ☐ Both

Statistic type for inference: *Specify voxel-wise or cluster-wise and report all relevant parameters for cluster-wise methods.*

(See Eklund et al. 2016)

Correction: *Describe the type of correction and how it is obtained for multiple comparisons (e.g. FWE, FDR, permutation or Monte Carlo).*

Models & analysis

n/a	Involved in the study	
☒	☐	Functional and/or effective connectivity
☐	☒	Graph analysis
☒	☐	Multivariate modeling or predictive analysis

Graph analysis: *Report the dependent variable and connectivity measure, specifying weighted graph or binarized graph, subject- or group-level, and the global and/or node summaries used (e.g. clustering coefficient, efficiency, etc.).*
